# Current Trends of Iridium‐Based Catalysts for Oxygen Evolution Reaction in Acidic Water Electrolysis

**DOI:** 10.1002/smsc.202300109

**Published:** 2023-11-27

**Authors:** Nguyen Thi Thu Thao, Jin Uk Jang, Arpan Kumar Nayak, HyukSu Han

**Affiliations:** ^1^ Department of Energy Engineering Konkuk University 120 Neungdongro Seoul 05029 Republic of Korea; ^2^ Department of Energy Science Sungkyunkwan University Suwon 16419 Republic of Korea

**Keywords:** acid media, adsorbate evolution mechanisms, iridium (Ir)-based catalysts, lattice oxygen-mediated mechanisms, oxygen evolution reaction

## Abstract

The proton exchange membrane water electrolysis (PEMWE) powered by renewable electricity offers a facile route for clean hydrogen production. The oxygen evolution reaction (OER) at the anode plays a major role in affecting the overall device efficiency due to its sluggish OER kinetics. Thus, it remains a challenge to develop robust and active catalysts for OER in acid media for efficient PEMWE. Currently, iridium (Ir)‐based materials, such as mono‐ and multimetallic Ir, Ir‐based oxides, pyrochlore iridate oxides, and Ir‐based perovskites, are the most promising OER catalysts in acid media for PEMWEs. Extensive research has been conducted to enhance the specific activity of Ir species to make cost‐effective. The present review aims to provide the recent progress on addressing the long‐term durability issue of Ir‐based catalyst for OER in acidic conditions, aspiring to inspire the researchers to design highly efficient and stable Ir‐based catalysts. Moreover, the detailed OER mechanism along with the dissolution nature of Ir species is discussed and summarized. Finally, the status, challenges, and prospects for the development of Ir‐based OER catalysts are discussed.

## Graphical abstract

In this mini review, recent progress of a comprehensive overview for the advancements and current application status of iridium‐based catalysts for proton exchange membrane water electrolyzer is summarized. The mini review introduces design strategies for anode catalysts with a potential to fulfill practical level of catalytic performance.

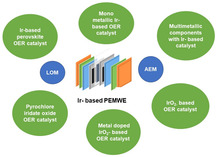

## 1 Introduction

Historically, carbon‐based fossil fuels have been considered as the primary global energy source. However, the combustion of these fuels has resulted in significant environmental problems such as air pollution, ecological destruction, global warming, etc.^[^ ^1^, ^2^ ^]^ As a result, there has been a significant focus on producing electrical power through environmentally friendly sources such as wind, solar, and hydropower. One major challenge of renewable energy sources is their intermittent nature, necessitating the development of energy storage and conversion technologies to enable on‐demand energy provision.^[^ ^3^ ^]^ One promising technique involves using hydrogen (H_2_) as an energy carrier to solve the future energy demand.^[^ ^4^ ^]^ Renewable energy‐derived electricity can be stored in H_2_ via electrochemical water splitting, and subsequently, electricity can be generated via the recombination of H_2_ with oxygen (O_2_).^[^ ^5^, ^6^, ^7^, ^8^ ^]^ The oxygen evolution reaction (OER), also known as the electrocatalytic oxidation of water, is a promising process in this area because of its accessibility and sustainability, with applications ranging from electricity storage to metal electrowinning.^[^ ^9^, ^10^ ^]^ However, the OER is a complicated multistep reaction that generates a considerable overpotential, which lowers the efficiency even when utilizing the standard catalysts that are now available.^[^ ^9^ ^]^ Further, the high electrode potentials during the OER make it challenging to maintain catalyst stability.

The different types of electrolysis for water splitting can be categorized based on the properties of the electrolyte used. The electrolytes used in water electrolysis can be classified into three main categories based on their properties: alkaline water electrolysis (AWE), solid oxide electrolysis (SOE), and proton exchange membrane (PEM) water electrolysis, respectively. Among the above categories, PEM electrolyzers are a promising technology for renewable electricity storage due to their numerous advantages over alkaline electrolyzers. These benefits include lower ohmic losses, higher voltage efficiency, purer gas, a more compact system design, higher current density, quicker response time, and a wider partial load range.^[^ ^11^, ^12^ ^]^ In addition to this, PEMs give excellent proton conductivity and offer the benefit of less gas crossover.^[^ ^11^ ^]^ However, the anodic OER in proton exchange membrane water electrolysis (PEMWE) suffers from sluggish kinetics due to the complex multiproton/electron‐coupled elementary steps, leading to high overpotential and reduced efficiency. Thus, it remains a challenge to develop robust OER electrocatalysts, which is critical for the practical implementation of PEMWE.

The increasing demand for clean and sustainable hydrogen production through PEMWE has motivated researchers to seek highly active, cost‐effective, and durable catalysts for efficient OER catalysis. However, the most abundant metal element in the Earth's crust is susceptible to corrosion in acidic OER environments. As a result, significant attention has been focused on investigating the catalytic properties of platinum group metal elements, known for their high resistance to acid.^[^ ^11^, ^13^, ^14^ ^]^ Extensive research efforts have revealed that critical factors such as composition and crystallinity play a vital role in influencing OER catalytic behavior.^[^ ^15^, ^16^, ^17^, ^18^ ^]^

In the present context, two types of oxide catalysts, iridium (Ir)‐based and Ru‐based, are seen as the most advanced catalysts for the OER in acidic conditions.^[^ ^19^, ^20^ ^]^ Though RuO_2_ exhibits higher catalytic activity, its lower stability than IrO_2_ limits its practical application.^[^ ^19^, ^21^, ^22^, ^23^ ^]^ The dissolution amount of RuO_2_ is found to be more than IrO_2_ in acid media, which makes IrO_2_ more stable than RuO_2_.^[^ ^18^ ^]^ In particular, Cherevko et al. observed that dissolution amount of RuO_2_ is 30 times higher than IrO_2_ under identical condition.^[^ ^24^ ^]^ In another study, Pedersen et al. found RuO_2_ shows a decrease in oxidation state beyond 1.5 V during OER, whereas no decrease in oxidation state observed for IrO_2_.^[^ ^19^ ^]^ Therefore, IrO_2_ has been widely utilized in operational PEM electrolysis systems. However, the extensive application of IrO_2_ is hindered by its limited availability.^[^ ^25^, ^26^ ^]^ Researchers have dedicated significant efforts to develop novel catalysts to replace IrO_2_ as an anode catalyst in PEMWE. However, these emerging catalysts often face challenges associated with detrimental structural changes during catalysis, including irreversible oxidation, surface reconstruction, and cation leaching, respectively. These procedures ultimately result in the breakdown of the crystal structure and the deterioration of the catalyst.^[^ ^27^, ^28^ ^]^ Furthermore, it is essential to recognize that the configuration and operating parameters of the three‐electrode setup and PEM electrolyzer significantly exhibit disparities, which can influence the performance of catalyst and suitability for PEMWE applications. As a result, despite the outstanding activity and stability demonstrated by anode catalysts, they often need to translate into high‐performance PEM electrolyzers effectively. This performance gap between active anode catalysts and the unique requirements of PEMWE applications highlights the need for most upcoming anode catalysts to meet these demands.^[^ ^29^ ^]^ Therefore, to investigate effective design strategies for new anode catalysts in PEMWE, it is crucial to create a comprehensive summary of potential catalysts and thoroughly examine the gap between these catalysts from the membrane electrode assembly (MEA). By doing so, researchers have the ability to improve the overall performance and efficiency of PEM electrolyzers, as well as bridge the existing performance gaps.

This review offers a comprehensive overview of the recent advancements and current application of Ir‐based catalysts in PEMWE. The article starts by providing an introduction to the stack structure and principles of PEM electrolyzers. Following that, we delve into the catalytic/degradation mechanisms observed during anode catalysis, which sheds light on how the catalysts function and degrade over time. Additionally, we engage in a discussion exploring the intriguing inverse relationship between the activity of the catalyst and its stability. This relationship is essential to understand as it impacts the long‐term performance of the PEM electrolyzer. The substantial efforts made in developing Ir‐based anode catalysts for PEMWE are retrospectively examined, emphasizing summarizing the representative candidates. We also compare the performance of the difference between effective anode catalysts and exceptional PEM electrolyzers. Comparing the PEM electrolyzer, which is utilized in industrial settings, to the three‐electrode cell configuration, which is used in laboratory evaluations, we investigate the differences between the two. To bridge the performance gap, our review suggests anode catalyst design and efficient assessment methods. Additionally, we present a perspective on the persisting obstacles and provide relevant recommendations to progress anode materials. These insights are expected to contribute significantly to promoting PEMWE hydrogen production technology.

## 2 Reaction Mechanisms

### 2.1 Structure and Principle of PEM Electrolyzers

**Figure 1:**
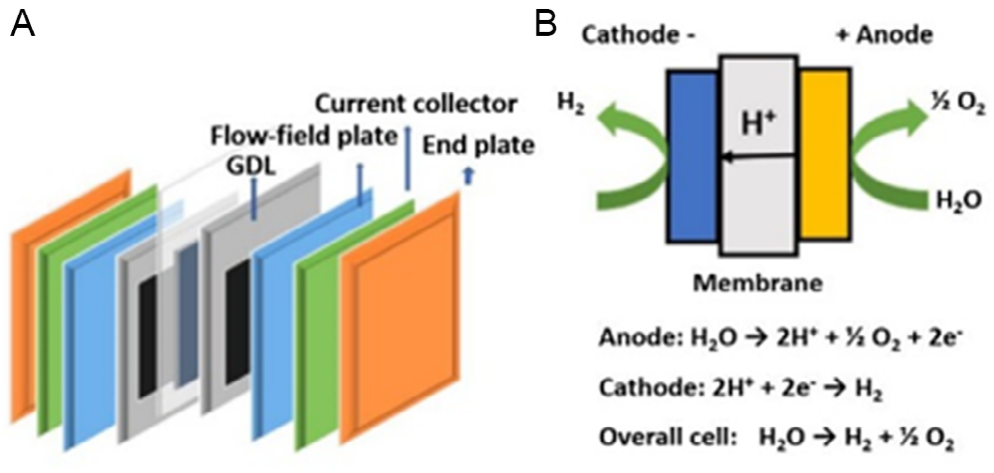
A) Structure; B) principle of the PEM electrolyzer.

PEM electrolysis is a promising technology for water splitting, and its cell components are similar to those of a PEM fuel cell. They both consist primarily of a PEM, a gas diffusion layer (GDL), a flow field, current collectors, and bipolar plates with flow channels (**Figure** **1**A). These parts play critical roles in facilitating the flow of electric charge and heat, as well as the movement of reactants and products. The bipolar plates and GDL assist in the movement of electric charge, thermal energy, and contain flow channels that allow for water supply and removal gas products. Additionally, a pure water supply system, water circulation pump, and heat exchange system are necessary to operate a PEM electrolyzer. These additional components ensure an adequate and continuous water supply and help manage the system's temperature. The HER and OER take place on the CCM (catalyst coated membrane) or GDE (gas diffusion electrode), which are coated with electrocatalysts.^[^ ^30^ ^]^ It is to be noted that noble‐metal free electrocatalysts are potential candidates for cathodic HER to replace Pt‐based catalysts.^[^ ^31^ ^]^ However, the anodic for OER catalyst continues to rely predominantly on rare and costly Ir oxides. Ir‐based catalysts constitute a significant portion of the total setup cost in the PEM electrolyzer system. On the other hand, the global Ir production is limited, posing a significant bottleneck for scaling up PEM‐based water electrolysis technologies. Because of its rarity and high cost, Ir can be a barrier to the broad adoption and commercialization of PEM electrolyzers. As a result, there is a need for alternative catalyst materials that are more common in order to overcome these obstacles.^[^ ^32^ ^]^ PEM electrolyzers need to have a lifespan exceeding 10 000 h for practical usage. Nevertheless, the acidic environment's electrochemical oxidation causes degradation of the anodic electrocatalyst, imposing limitations on its overall lifetime.^[^ ^11^ ^]^ Therefore, an active and durable OER catalysts is crucial for the overall energy efficiency of the PEM electrolyzer.^[^ ^33^ ^]^ In PEM water electrolysis, the process involves the electrochemical splitting of water into hydrogen and oxygen, occurring at the cathode and anode electrodes, respectively. During PEM water electrolysis, water is supplied to the anode, where it undergoes splitting into oxygen (O_2_), protons (H^+^), and electrons (e^−^). The protons then migrate through a proton‐conducting membrane to reach the cathode side. Simultaneously, the electrons flow out of the anode through the external power circuit, which generates the driving force (cell voltage) for the reaction. At the cathode, the protons and electrons recombine, leading to the production of hydrogen. The detailed mechanism process is depicted in Figure 1B.

### 2.2 Mechanism of OER

The two most widely recognized mechanisms for the OER are the adsorbate evolution mechanism (AEM) and the lattice oxygen evolution mechanism (LOM). The AEM and LOM are discussed in detail, and recent experimental findings are presented to provide a comprehensive understanding of these mechanisms. The AEM is a commonly studied mechanism for the OER, where the intrinsic activity of the catalyst is primarily determined by the binding strengths with intermediates. In electrocatalytic processes, balancing the binding strength between active sites and intermediates is crucial for optimal activity.^[^ ^34^ ^]^ In contrast, the LOM involves the oxidation of the catalyst's lattice oxygen to release oxygen and participate in the OER reaction. This mechanism can provide insights into the remarkable catalytic performance of solid‐phase catalysts, and a deeper understanding of the reaction mechanism and factors can guide the development of more effective electrocatalysts for OER.

#### 2.2.1 AEM

Following the Sabatier principle in acidic environments, the AEM entails a sequence of four successive proton–electron transfer reactions via OH*, O*, and OOH* intermediates. This procedure converts two adsorbed water molecules at the active site into oxygen.^[^ ^35^, ^36^ ^]^ The AEM mechanism involves several steps. The main mechanisms discussed above can be summarized as follows in **Table** **1**.

**Table 1:** The mechanistic models of AEM.^[^ ^14^, ^132^ ^]^

	Mechanism in acid pathway
Oxide path	H_2_O + M→M─OH + H^+^ + e^−^ 2M─OH→M─O + M + H_2_O 2M─O →2M + O_2_
Electrochemical oxide path	H_2_O + M → M─OH + H^+^ + e^-^ M─OH→M─O + H^+^ + e^-^ 2M─O→2M + O_2_
Electrochemical metal peroxide path	H_2_O + M→M─OH + H^+^ + e^−^ 2M─OH→M─O + M + H_2_O M─O + H_2_O→M─OOH + H^+^ + e^-^ 2M─OOH→M─O + H_2_O + O_2_ + M

**Figure 2:**
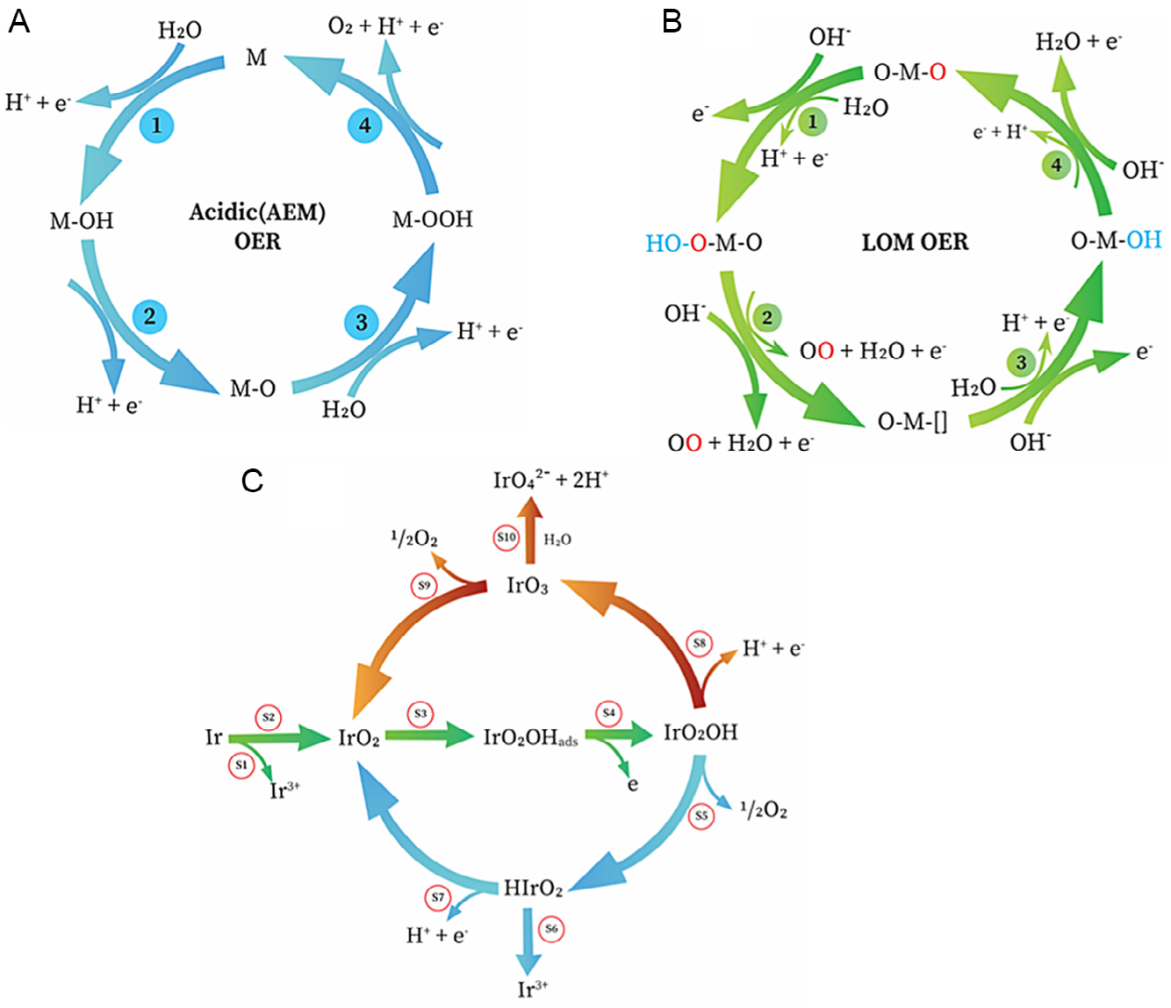
Schematic illustration of the A) AEM, B) LOM, and C) mechanism connecting the OER to potential routes for Ir dissolution.

Initially, water molecules bind to metal (M) sites on the material's surface through a single‐electron process, resulting in the formation of an *OH molecule at the M site. Subsequently, the *OH molecule undergoes oxidation and transforms into a *O molecule. Another water molecule then attaches to the *O molecule, forming an intermediate *OOH species. Finally, the *OOH species undergoes an additional oxidation step, leading to the release of molecular oxygen and the restoration of the original M site (**Figure** **2**A).

The activity of the OER is influenced by the reaction energetics of each elementary step, which are dependent on the binding energies between OH*, O*, and OOH* intermediates. Among these steps, the one with the highest change in free energy (Δ*G*) is considered the rate‐limiting step (RLS), dictating the overall performance of the OER. Notably, there exists a linear correlation between the adsorption energies of OH*, O*, and OOH* intermediates. In most OER reactions, either the deprotonation of OH* or the formation of OOH* can act as the RLS. To predict the activity of OER catalysts, a descriptor known as the difference in binding energy between O* and OH* intermediates (Δ*G*_O*_ − Δ*G*_OH*)_ is introduced. This descriptor serves as a valuable tool for evaluating catalyst performance and predicting activity in the OER process.^[^ ^16^ ^]^ According to Sabatier's principle, in the case of an optimal catalyst, the oxygen‐binding energy should be moderate, avoiding extremes of strength or weakness. The difference between Δ*G*_O*_ and Δ*G*_OH*_ can be used to estimate the oxygen binding capacity, offering a reasonable assessment of catalytic effectiveness.

In the case of Ir‐based catalysts, the active site is commonly identified as the coordinated unsaturated Ir ion. It has been widely observed that the initial low valence state of Ir ions is widely noted for weakening Ir–O binding, enhancing catalytic activity.^[^ ^37^ ^]^ This result is in line with earlier experimental research, such as that of Shao et al., who established that the lower valence Ir site in 1T–IrO_2_ acts as the active site capable of modifying the binding energy of hydroxyl groups.^[^ ^38^ ^]^ In another study, Nong et al.^[^ ^39^ ^]^ made an interesting discovery regarding the IrNiO_*x*_ catalyst. They observed that the presence of low valence Ir species in the catalyst led to an increased number of surface *d*‐band holes in Ir, particularly after the leaching of Ni. Additionally, they found that the in situ oxidation of Ir to a high‐valence state was responsible for the catalyst's high OER activity. The researchers emphasized the significance of the initial valence state of Ir, noting that lower valence Ir species were more conducive to the formation of active sites with high‐valence Ir, thereby contributing to enhanced OER performance. However, the AEM has limitations in fully explaining the catalytic activity. Theoretical studies have revealed a proportional connection between OH* and OOH*, where Δ*G*_OOH*_ = Δ*G*_OH*_ + 3.2 ± 0.2 eV. This implies that the water oxidation procedure would demand a greater theoretical overpotential of 370 mV. However, this theoretical prediction does not entirely explain the observed mechanisms in numerous highly efficient acidic OER catalysts.^[^ ^16^, ^17^ ^]^ Furthermore, the AEM suggests relative stability of the catalyst structure. However, numerous studies have highlighted significant changes in the structure and composition of catalysts during the reaction. As a result, relying solely on the AEM is insufficient for predicting the properties of OER catalysts.

#### 2.2.2 LOM

The conventional OER mechanism, AEM, has limitations of scaling relationship among intermediate oxygen molecules, resulting in high theoretical overpotentials. To overcome this, effective strategies have been adopted, but a new mechanism known as LOM has emerged as a more widely accepted approach.^[^ ^36^ ^]^ LOM does not depend on the binding energy of intermediates for assessing OER activity. However, LOM follows the initial steps as similar to AEM mechanism, where an *O species is formed through the first two steps (Equation (1) and (2)). The *O species can then combine with oxygen ions in the catalyst's lattice structure, resulting in the release of oxygen molecules and form abundant oxygen vacancies V_o_ (Equation (3)). These vacancies can be filled by a water molecule, resulting in the formation of an *OH species (Equation (4)). Finally, the proton from the *OH species is removed through a one‐electron oxidation step (Equation (5)).^[^ ^40^, ^41^ ^]^ The proportional correlation among *OH and *OOH is not considered in LOM, resulting high coverage of intermediates is not required and the oxygen within the catalyst lattice acts as the intermediary.^[^ ^42^, ^43^ ^]^ LOM favorable electrocatalysts possess high TM—O bond covalency, enabling high intrinsic reactivity and surface reformation.^[^ ^44^ ^]^ In situ ^18^O isotope labeling mass spectrometry is widely used to study the role of lattice oxygen and active oxygen species. The LOM process in acidic environment can be concluded as follows:
(1)

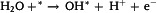

(2) $${\text{OH}}^{*}\to O^{*}\;+{\text{ H}}^{+}+{\text{ e}}^{-}$$

(3) $$O^{*}\;+{\text{ O}}_{L}\to {\text{ O}}_{2}+{\text{ V}}_{o}$$

(4) $$V_{o}+{\text{ H}}_{2}\text{O }\to {\text{ OH}}^{*}\;+{\text{ H}}^{+}+{\text{ e}}^{-}$$

(5)

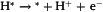




Several notable studies have contributed to advancing the understanding of the LOM in acidic conditions.^[^ ^45^, ^46^ ^]^ Tarascon and co‐workers have reported a modified OER mechanism on La_2_LiIrO_6_ in an acidic environment.^[^ ^46^ ^]^ This mechanism involves the initial step of delithiation and surface oxidation of Ir (4^+^ → 5^+^). The essential connection of O—O bonds can occur via two distinct pathways in acidic environments: one involves water attack followed by deprotonation, while the other involves the direct connection of two Ir^VI^–O species. Once oxygen is released, the created oxygen vacancies can be regenerated through either water dissociation or transfer of lattice oxygen from the bulk material. These findings provide valuable insights into the intricate LOM processes in acidic environments. Cherevko and co‐workers proposed a modified approach to simplify the mechanism and prevent overcrowding, combining the nucleophilic attack and proton removal steps into a single reaction.^[^ ^45^ ^]^ The lattice oxygen is the pivotal site, influenced by the oxygen–oxygen mechanism (OOM). This involves a sequence of consecutive stages, including activating the lattice oxygen, removing oxygen, and restoring vacancies through water adsorption. This completes the entirety of the OER cycle. Nevertheless, incorporating lattice oxygen into the reaction poses a significant risk in terms of sacrificing stability. Additionally, the crystalline structure of IrO_*x*_ also plays a crucial role in determining the stability. Notably, they observed that the involvement of lattice oxygen in amorphous Ir oxide results in a substantial reduction in catalyst stability.

### 2.3 Stability of Catalysts during Acidic Oxygen Evolution Reaction

The practical usability of proton exchange membrane water electrolyzers heavily relies on the extended‐term durability of their electrocatalysts. Researchers employ two popular methods to assess electrocatalyst stability. The first method involves recording chronopotentiometry or chronoamperometry curves for extended periods, typically over 12 h. Any increase in overpotential at a constant current density or a decline in current density at a steady potential indicates poor catalyst durability. The second method, the accelerated durability test, entails conducting cyclic voltammetry tests at a fast scanning rate (e.g., 50–200 mVs^−1^) for numerous cyclic voltammetry cycles and within a typical potential range (e.g., 1.4–1.8 V_RHE_).^[^ ^47^ ^]^ To assess the stability of the catalyst, researchers compare the shift in overpotential at a particular current density before and after the cycling test. A minor change in overpotential indicates better stability as an OER catalyst. Additionally, researchers employ various techniques, such as measuring catalyst dissolution, to investigate the structural stability of these catalysts. Noble metals such as Ru, Ir, Rh, Pd, Pt, and Au have been found to experience both transient and equilibrium dissolution during the OER processes.

In redox reactions involving surface oxides, transient dissolution can occur, which is a temporary catalyst dissolution. On the other hand, the continuity of dissolution in a stable state depends on the distinct OER mechanism occurring on the metal surface. Nanomaterials (NMs) like ruthenium (Ru) and gold (Au), which follow the LOM, demonstrate a low Tafel slope but also exhibit a higher rate of catalyst dissolution.

On the other hand, NMs catalysts such as platinum (Pt) and palladium (Pd) that follow the AEM in the OER processes show high Tafel slopes (indicative of slower catalytic activity) and a slower rate of metal leaching (lower dissolution rate). The relationship between activity and durability has been a critical focus of the study. OER catalysts with similar structures exhibit opposite trends in their activity–stability trade‐offs. In other words, stimuli that are highly active tend to be less stable, while more stable catalysts may have lower activity. As a result, selecting OER catalysts that strike a balanced compromise between activity and stability becomes crucial in practical applications. A suitable catalyst should have sufficient training to drive the desired reaction effectively while maintaining long‐term stability to avoid rapid degradation or loss of catalytic performance. Achieving this balance is essential for optimizing electrochemical devices’ overall performance and efficiency, such as fuel cells or water electrolyzers, which rely on OER catalysts.^[^ ^48^, ^49^, ^50^, ^51^ ^]^ However, recent studies have showcased promising results, demonstrating satisfactory activity and stability simultaneously, mainly using Ir‐based catalysts, which will be discussed in subsequent sections of the article.

### 2.4 Rational Design of OER Electrocatalysts

The structural design of Ir‐based catalyst is a challenging task for stable acidic OER. Besides, there are two main categories of Ir‐based catalysts utilized in acidic OER: Ir oxide and Ir (0‐valent).

Commercial rutile IrO_2_ exhibits relatively low catalytic activity in acidic environments, reducing energy conversion efficiency. In contrast, amorphous Ir oxide (IrO_*x*_) exhibits superior catalytic activity than crystalline IrO_2_, attributed to its higher number of active sites and more flexible structures.^[^ ^52^, ^53^ ^]^ Moreover, amorphous Ir oxide possesses two binding energies associated with Ir species,^[^ ^54^, ^55^ ^]^ one corresponding to Ir(IV) in rutile IrO_2_^[^ ^54^ ^]^ and the other associated with the formation of chemoelectrophilic oxygen O^I−^ species induced by the higher binding energy Ir(III) species. This leads to the production of O^I−^ species and may exacerbate the nucleophilic attack in the OER procedure.^[^ ^54^ ^]^ Studies conducted by Mayrhofer and co‐workers^[^ ^56^ ^]^ indicate that the existence of hydrated Ir oxide with Ir (III) content encourages the formation of lattice oxygen precipitates, leading to an acceleration of Ir dissolution. On the other hand, Jones et al. suggest that the crystallinity of Ir oxide can affect its activity and stability.^[^ ^57^ ^]^ IrO_2_ with well‐defined crystalline structure experiences deprotonation mainly on its surface, whereas amorphous and poorly crystalline IrO_*x*_ tend to undergo deprotonation in the nearby surface region,^[^ ^57^ ^]^ reducing the barriers to OER via electron doping impacts by encouraging the Ir dissolution.^[^ ^57^ ^]^

During conditions of acidic OER, the surface of Ir undergoes oxidation to form IrO_*x*_ species, which facilitates the continuation of the reaction.^[^ ^58^ ^]^ The Ir oxide formed electrochemically exhibits higher solubility due to poor thermodynamic stability.^[^ ^24^ ^]^ The dissolution of Ir (0) can occur through two possible routes. One route involves direct dissolution, where Ir(0) is directly transformed into to Ir(III). The other route involves a two‐step process, where Ir(0) is first oxidized to Ir(V) and then subsequently reduced to Ir(III).^[^ ^59^ ^]^ The creation of Ir oxide species is not dependent on the overall surface free energy.^[^ ^60^ ^]^ Further the lower surface energy of Ir(210) crystal surfaces and its initial level leads to the creation of Ir(IV) oxide species. This process leads to a reduction in the electrochemically active surface area and, consequently, a decrease in catalytic activity. In an acidic environment, the surface condition of the catalyst can undergo significant changes following the OER procedure. As a result, there might be a weak correlation between the surface on the catalyst and its OER performance. Researchers have demonstrated that the outcomes resulting from the electrochemical oxidation of Ir, conducted on distinct crystal plane orientations, may exhibit variations. This implies that the crystal plane orientation of the catalyst could impact the OER reaction and the formation of products. This underscores the complexity of the OER process and the importance of considering various factors that can impact the catalyst's performance in acidic environments. Li et al.^[^ ^61^ ^]^ Ir(024) high‐index open planes form metastable metastoichiometric IrO_*x*_ species in the subsurface with a higher performance. In contrast, topmost closed planes like Ir(113) transform into more stable oxides like IrO_2_, which could reduce catalytic activity.

In the context of acid OER, Ir‐based oxide catalysts have been widely utilized. Recent studies have shown a strong correlation between Ir dissolution and OER.^[^ ^59^ ^]^ Depending on the applied potential, Ir electrodes undergo the OER catalytic cycle primarily through either Ir^III^ or Ir^VI^, leading to different dissolution pathways (Figure 2C). For heat‐treated Ir oxide with lower activity, a higher applied potential is required. This elevated potential facilitates the further oxidation of the core Ir^V^ intermediate into volatile IrO_3_. IrO_3_ can then undergo decomposition to form IrO_2_ or dissolve into soluble IrO_4_^2−^. On the other hand, highly efficient metallic Ir and Ir oxide deposited through reactive sputtering demonstrate a substantial reaction at a low overpotential during the OER. However, the potential is not sufficient to oxidize Ir^V^. Consequently, the Ir^V^ intermediates decompose into HIrO_2_ species, which can then dissolve into soluble Ir^III^ ions or deprotonate to form IrO_2_. As mentioned earlier, the OER and the dissolution of Ir are interconnected processes that involve a common unstable intermediate. Consequently, achieving a highly stable catalyst requires effective inhibition of the dissolution of these intermediates. However, the kinetics of the reactions involved influences the competition between different dissolution or deposition pathways. Thus, a deeper understanding of the activity–stability relationship in actual catalysts necessitates more comprehensive investigations involving kinetic simulations and advanced characterization technique.

## 3 Ir‐Advanced in Anode Catalyst for PEMWE

### 3.1 Monometallic Ir‐Based OER Catalyst

**Figure 3:**
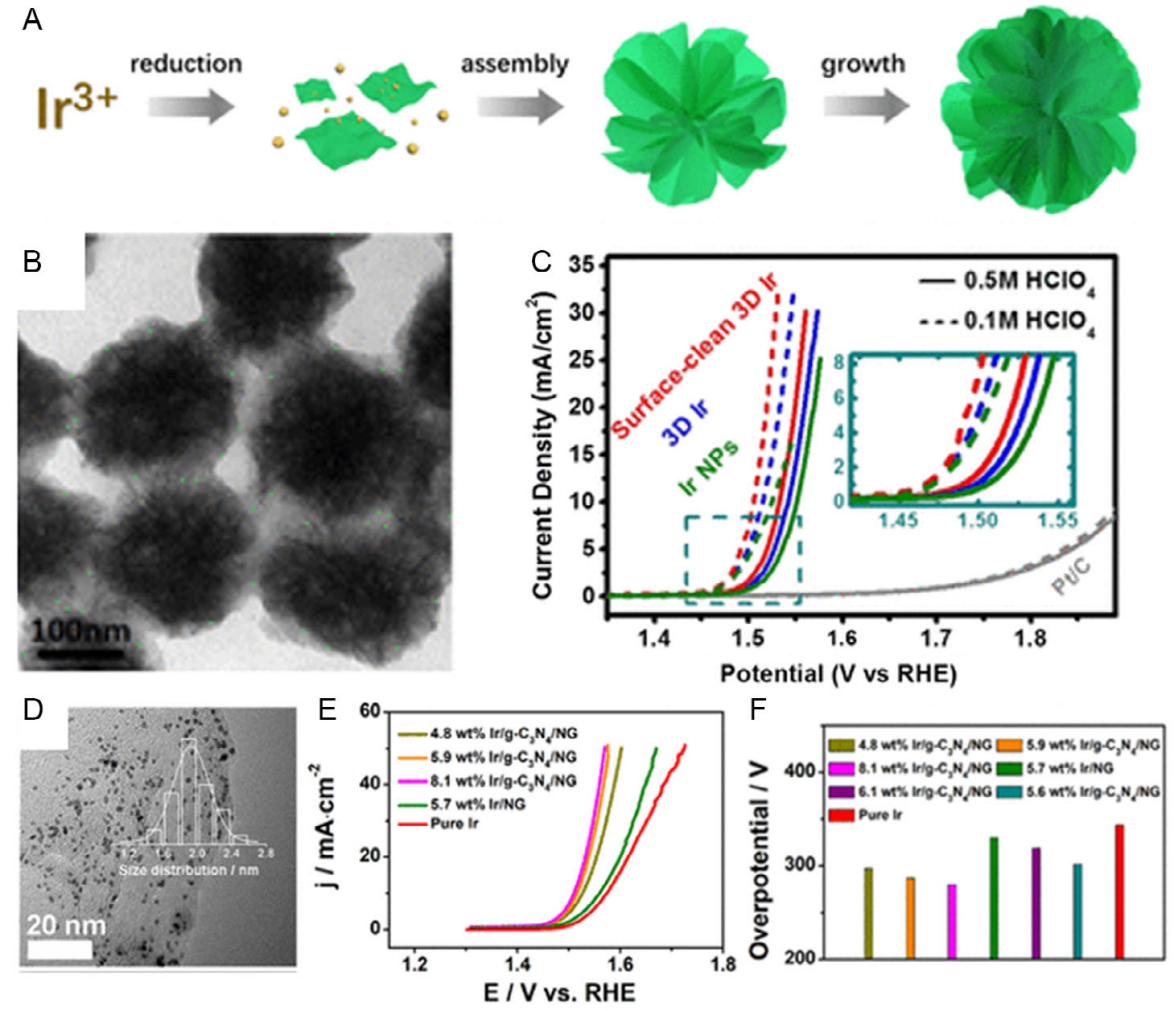
A) A diagram depicting the fabrication process of 3D Ir superstructures. B) HAADF–STEM images of 3D Ir superstructures. C) OER polarization curves of the Ir catalyst and Pt/C in HClO_4_ electrolyte.^[^ ^70^ ^]^ (A,B,C) Reproduced with permission.^[^ ^60^ ^]^ Copyright 2016, American Chemical Society. D) 5.9 wt% Ir/g‐C_3_N_4_/NG TEM image. E) OER performance and F) overpotential at 330 mV of as‐prepared catalysts.^[^ ^72^ ^]^ (D,E,F) Reproduced with permission.^[^ ^61^ ^]^ Copyright 2018, American Chemical Society.

Though Ir is found to be slightly less activity than Ru, yet the excellent durability and low dissolution of Ir in acidic conditions make it a valuable electrocatalyst for the OER.^[^ ^62^, ^63^, ^64^, ^65^, ^66^, ^67^, ^68^, ^69^ ^]^ As a result, great efforts have been invested in enhancing Ir‐based materials by employing diverse nanostructures and compositions to enhance the catalytic effective in OER. To address this issue, Huang and co‐workers^[^ ^70^ ^]^ reported the first instance of a successful wet‐chemical method to create a 3D Ir superstructure using ultrathin Ir nanosheets as subunits. This unique superstructure with 3D accessible sites, maximized surface area, and optimal layer distance has the potential to improve electrochemical energy conversion significantly (**Figure** **3**A). This novel structure demonstrated outstanding OER performance in acidic and alkaline conditions. The 3D superstructure mentioned the 3D Ir superstructure initially appears nearly spherical, with monodisperse nanospheres, each nanosphere is found to be a 3D structure consisting of multiple building blocks of ultrathin nanosheets. The ultrathin nanosheets measure <1.5 nm and comprise only several layers of Ir, as observed through high angle annular dark field scanning transmission electron microscopy (HAADF–STEM) imaging (Figure 3B). This design allows for a highly exposed active surface area, leading to outstanding OER performance in acidic electrolytes. The 3D Ir and surface‐clean 3D Ir superstructures exhibit low onset potentials of approximately 1.45 V_RHE_ in 0.1 M HClO_4_ and approximately 1.47 V_RHE_ in 0.5 M HClO_4_ (Figure 3C).Tackett and co‐workers devised a distinctive core–shell structure characterized by an Ir/metal nitride morphology. This specific architecture led to a notable enhancement in the mass activity of Ir compared to commercial IrO_2_, exhibiting a twofold increase.^[^ ^71^ ^]^ Additionally, Shao et al. presented a simple method to synthesize ultrasmall Ir nanoparticles supported on g‐C_3_N_4_/nitrogen‐doped graphene (g‐C_3_N_4_/NG; Figure 3D). The sample 5.9 wt% Ir/g‐C_3_N_4_/NG displayed the largest OER increase by utilizing the synergistic effect among the different catalyst components, at just 287 mV overpotential to achieve a current density of 10 mA cm^−2^. According to calculations, the mass activity of the 5.9 wt% Ir/g‐C_3_N_4_/NG catalyst was 2.31 A mgIr^−1^, which was 2.8 and 4.4 times greater than that of the 5.7 wt% Ir/NG (0.81 A mgIr^−1^) and pure Ir (0.52 A mgIr^−1^), respectively. These findings demonstrate the potential of Ir/g‐C_3_N_4_/NG catalyst for effective OER applications (Figure 3E,F).^[^ ^72^ ^]^ In recent studies, the utilization of Ir single‐atom catalysts (SACs) has extended to the field of acidic OER. Notably, Luo et al. developed a double protective strategy to enhance the stability and performance of Ir SACs for acidic water electrolysis. This approach involved dispersing Ir atoms in/onto Fe nanoparticles and incorporating IrFe nanoparticles into nitrogen‐doped carbon nanotubes, resulting in the formation of Ir‐SA@Fe@NCNT catalysts. When employed as an acidic OER catalyst with a remarkably low Ir content (approximately 1.14 μg cm^−2^), Ir‐SA@Fe@NCNT exhibited impressive electrochemical performance. It required a small overpotential of 250 mV to achieve a current density of 10 mA cm^−2^ in 0.5 m H_2_SO_4_ solutions. Remarkably, a high mass activity was observed at 270 mV overpotential, which is 1370‐fold higher than that of commercial IrO_2_.^[^ ^73^ ^]^ Oh et al. synthesized Ir nanodendrites supported on antimony‐doped tin oxide (ATO) and employed in single‐cell PEM electrolyzer exhibiting a current density of 1.5 mA cm^−2^ at 1.8 V cell voltage with excellent durability under constant current.^[^ ^74^ ^]^

### 3.2 Multimettalic Components with Ir‐Based OER Catalyst

**Figure 4:**
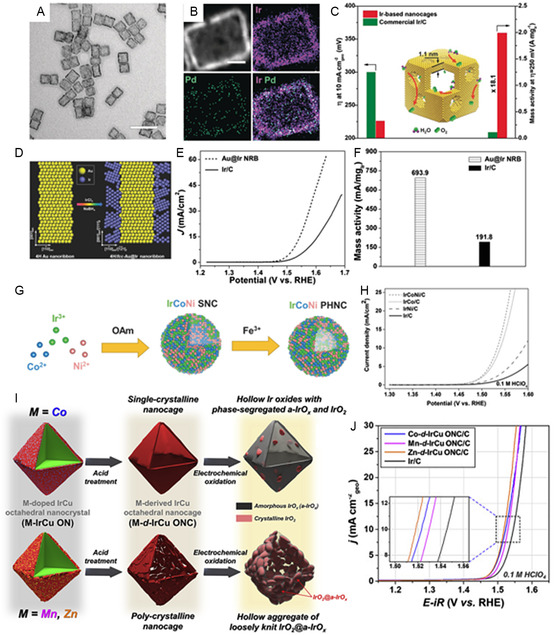
A) TEM and B) HAADF–STEM image. C) Overpotential at 10 mA cm^−2^ and mass activities at 250 mV of Ir‐based nanocages.^[^ ^82^ ^]^ (A,B,C) Reproduced with permission.^[^ ^79^ ^]^ Copyright 2019, Wiley. D) Schematic illustration for the seed‐mediated synthesis of 4H/fcc‐Au@Ir core–shell NRBs. E) Polarization curves. F) Mass activities at potential of 1.55 V versus RHE H_2_SO_4_ 0.5M.^[^ ^83^ ^]^ (D,E,F) Reproduced with permission.^[^ ^80^ ^]^ Copyright 2016, Wiley. G) Schematic illustration of the formation process of IrCoNi PHNCs. H)Polarization curves for OER in 0.1M HclO_4_.^[^ ^84^ ^]^ (G,H) Reproduced with permission.^[^ ^81^ ^]^ Copyright 2017, Wiley. I) A schematic illustration of the dopant‐assisted strategy to create single‐crystalline and polycrystalline Ir‐based ternary nanocages, along with their structural transformation after OER. J) OER polarization of M–*d*–IrCu ONC/C (M=Co, Mn, or Zn) in 0.1 M HClO_4_.^[^ ^86^ ^]^ (I,J) Reproduced with permission.^[^ ^83^ ^]^ Copyright 2020, Elsevier.

Although monometallic Ir nanostructures have demonstrated some improvement in the performance of the OER, their activity and stability still remain unsatisfactory. Previous studies have demonstrated that the reported Ir nanostructures exhibit OER stability of less than 10 h.^[^ ^70^ ^]^ Due to the limited accessibility and outstanding characteristics of Ir, extensive efforts have been dedicated to the efficiency of the OER through a variety of approaches.^[^ ^75^, ^76^, ^77^ ^]^One commonly employed and widely acknowledged strategy involves designing and synthesizing multimetallic nanostructures based on Ir by combining Ir with less valuable transition metals such as Pd, Au, Ni, Co, and Fe through alloying.^[^ ^78^, ^79^, ^80^ ^]^ In comparison to pure Ir and commercial IrO_2_, the activity and durability of Ir‐based nanostructures can be dramatically enhanced by synthesizing the catalyst through careful alloying and composition optimization.^[^ ^80^, ^81^ ^]^ Significant modification of the Ir electronic structure by alloying results in optimized adsorption and desorption processes of adsorbates on the catalyst surface, which improves OER performance and durability. Consequently, the observed OER activity was significantly enhanced. Additionally, the introduction of foreign atoms serves the purpose of reducing the reliance on scarce and precious metals. In particular, Xia et al. developed cubic nanocages with a Pd@Ir core–shell structure having an average edge length of 19.7 ± 3.1 nm in two steps: first, the creation of Pd@Ir core–shell nanocubes by depositing Ir atoms conformally on Pd nanocubes with controlled edge length; and second, the selective removal of Pd cores through chemical etching (**Figure** **4**A,B). These Pd@Ir core–shell nanocages exhibited an outstanding mass activity as an OER catalyst, reaching 18.1‐fold higher performance compared to the commercial Ir/C at 250 mV overpotential (Figure 4C).^[^ ^82^ ^]^ Likewise, Zhang et al. demonstrated the formation of 4H/fcc‐Au@Ir core–shell nanoribbons (NRBs) through direct growth of Ir on 4H Au NRBs under ambient conditions (Figure 4D). Remarkably, the 4H/fcc‐Au@Ir core–shell NRBs demonstrate outstanding electrocatalytic activity for the OER under acidic conditions, significantly outperforming the commercial Ir/C catalyst in a H_2_SO_4_ solution. Au@Ir NRBs show a lower onset potential, a lower potential, and the mass activity is 3.6 times greater than commercial Ir/C (Figure 4E,F).^[^ ^83^ ^]^ Guo et al. reported a versatile approach for controllable synthesis of IrM (M = Co, Ni, CoNi) multimetallic porous hollow nanocrystals (PHNCs). The method involves etching Ir‐based, multimetallic, solid nanocrystals using Fe^3+^ ions as catalysts to enhance water‐splitting efficiency.^[^ ^84^ ^]^ The synthesis of IrM PHNCs involved a two‐step process, starting with the preparation of IrM SNCs in oleylamine (OAm) at a high temperature, followed by etching with Fe^3+^ ions in a hexane/ethanol mixture at room temperature. The resulting IrCoNi PHNCs exhibited monodispersity and high porosity, as shown in Figure 4G. Density functional theory (DFT) simulations revealed that the ligand effect of alloying caused a shift in the density of states (DOS) away from the Fermi level, resulting in a corresponding shift of the *d*‐band center away from the Fermi level. This shift weakened the adsorption of oxygen intermediates and enhanced the catalytic properties. The alloying‐induced decrease in activation energy significantly improved the IrM PHNCs catalyst activity during OER performance in acidic electrolytes. In 0.1 M HClO_4_, the catalyst only requires a 303 mV overpotential to attain a current density of 10 mA cm^−2^, demonstrating excellent OER activity in acidic environment (Figure 4H). Guo et al. accomplished an equilibrium between controlling adsorption energy and resisting corrosion by designing alloy Ir–W porous nanodendrites that exhibited high activity and outstanding stability in acidic conditions.^[^ ^85^ ^]^ At 300 mV overpotential, the current density of Ir–W nanodendrites was 3 times that of Ir/C, and they maintained outstanding durability after 3000 cycles in 0.1 M HClO_4_. In addition, it was discovered that IrNi and IrCo nanoflowers substantially improved the OER performance and stability in 0.1 M HClO_4_.^[^ ^81^ ^]^ Lee et al.^[^ ^86^ ^]^ conducted a synthesis of hollow octahedral nanocrystals, denoted as M‐doped IrCu ONs (M = Co, Mn, or Zn), with each dopant resulting in different structural characteristics (Figure 4I). When doped with Mn or Zn, a polycrystalline shell composed of Ir‐based material was obtained, whereas Co dopant led to the formation of a single‐crystalline shell. During the OER, the Mn–IrCu and Zn–IrCu ONs underwent transformation into loosely knit crystalline domains consisting of IrO_2_ and amorphous a‐IrO_*x*_. On the other hand, the Co–IrCu ONs transformed into a random mixture of a‐IrO_*x*_ and IrO_2_ phases. Notably, the loosely knit crystalline domains of IrO_2_@a‐IrO_*x*_ exhibited significantly enhanced stability during the OER compared to the randomly mixed a‐IrO_*x*_ and IrO_2_ phases (Figure 4J). The enhanced stability was attributed to the unique structural arrangement, characterized by stronger interconnection between [IrO_6_] octahedral units and faster charge transfer between crystalline IrO_2_ and a‐IrO_*x*_ within the loosely knit crystalline domains of IrO_2_@a‐IrO_*x*_. Yin et al. presented coelectrodeposition to create Ir‐SACs (Ir single‐atoms catalyst) coupling with oxygen vacancies on ultrathin NiCo_2_O_4_ porous nanosheets and exhibit outstanding OER performance with an ultralow overpotential of 240 mV at *j* = 10 mA cm^−2^ and long‐term stability of 70 h in acid solutions.^[^ ^87^ ^]^

A dandelion spore‐structured self‐supporting IrNi was proposed as a bifunctional catalyst for PEMWE by Yeo et al.^[^ ^88^ ^]^ The dealloyed nanoporous IrNi (DNP‐IrNi) was created by H‐induced coelectrodeposition with a constant current during the dealloying process. The DNP‐IrNi catalyst maintained activity for at least 50 h at 200 mA cm^−2^ and had a small OER overpotential of 248 mV at 10 mA cm^−2^. The DNP‐IrNi was used as a bifunctional catalyst in PEMWE single cell and observed remarkable performance of 6.5 Acm^−2^ at a cell voltage of 2.0 V with a degradation rate of only1.58 mV h^−1^ after 100 h stability test at 2 A cm^−2^ by applying 0.67 mgIr cm^−2^ to both electrodes.^[^ ^88^ ^]^

### 3.3 IrO_2_‐Based OER Catalyst

**Figure 5:**
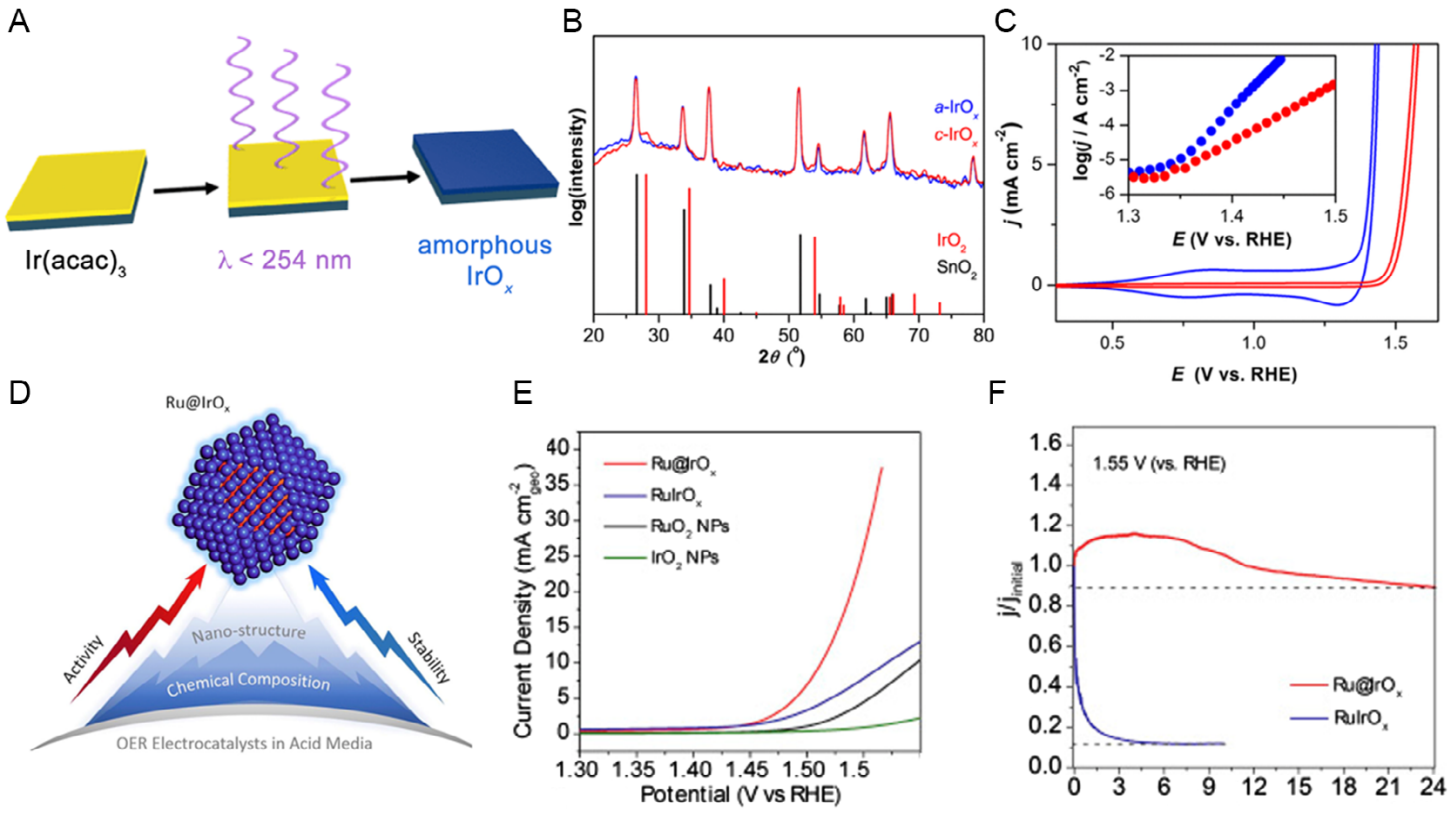
A) Illustration depicting the process of creating amorphous IrO_*x*_. B) XRD patterns for α‐IrO_*x*_ and c‐IrO_*x*_. C) Cyclic voltammograms (CVs) recorded on film of α‐IrO_*x*_ and c‐IrO_*x*_ in 1.0 M H_2_SO_4_.^[^ ^89^ ^]^ (A,B,C) Reproduced with permission.^[^ ^84^ ^]^ Copyright 2014, American Chemical Society. D) Scheme of Ru@IrO_*x*_ core–shell nanocrystals. E) Linear sweep voltammogram (LSV) curves in 0.05M H_2_SO_4_. F) Current–time chronoamperometric response of Ru@IrO_*x*_ and RuIrO_*x*_ electrocatalysts at 1.55 V.^[^ ^100^ ^]^ (D,E,F) Reproduced with permission.^[^ ^95^ ^]^ Copyright 2019, Elsevier.

Apart from Ir metallic, their respective oxides have also shown remarkable OER activity in acidic environments, leading to the development of highly efficient IrO_2_‐based catalysts. Several strategies have been pursued in recent decades to enhance the activity of these oxides. Among several strategies, designing amorphous nanostructures plays a crucial role in enhancing the OER activity and stability. In general, amorphous materials have demonstrated superior catalytic activity compared to their crystalline counterparts. Extensive research has focused on synthesizing various types of amorphous nanostructures. For instance, Smith et al. synthesized amorphous Ir oxide (α‐IrO_*x*_) films using acetylacetonate precursor via light‐induced decomposition method in an ambient air atmosphere (**Figure** **5**A,B). These α‐IrO_*x*_ films display excellent electrocatalytic properties for the OER in acidic media. Notably, this method offers a cost‐effective and scalable approach for the deposition of α (alpha)‐IrO_*x*_ on electrode surfaces, and it showed a small overpotential of 220 V at 10 mA cm^−2^ (Figure 5C).^[^ ^89^ ^]^ Xing et al. demonstrated an ammonia‐induced pore‐forming method that produced IrO_2_ micro/mesoporous catalyst shows excellent specific surface area of 363.3 m^2^ g^−1^. The structure of the catalyst resulted in substantially lower charge transfer resistance, lower overpotential, and greater durability for acidic OER activity.^[^ ^90^ ^]^ Efforts have been made to control the morphology of IrO_2_ to enhance its OER activity. Various strategies have been explored, including the synthesis of ultrathin IrO_2_ nanoneedles,^[^ ^91^ ^]^ aligned IrO_2_ nanotube arrays,^[^ ^92^ ^]^ ultraporous IrO_2_ hierarchical structures,^[^ ^93^ ^]^ IrO_*x*_ nanosheets,^[^ ^94^, ^95^ ^]^ and 3D ordered macroporous IrO_2_.^[^ ^96^ ^]^ These morphological modifications aim to optimize the catalytic performance of IrO_2_ in OER processes. Weber et al. conducted microscopic studies on potential‐induced corrosion of IrO_2_ (110) thin films.^[^ ^97^, ^98^ ^]^ Their investigations revealed that pitting corrosion initiated at the surface grain boundaries of IrO_2_ films when the applied potential reached 1.48 V_RHE_. Xu et al.^[^ ^99^ ^]^ also reported pitting corrosion of Ir‐based catalysts during acid‐driven OER processes. Furthermore, metallic Ru or RuO_2_ has been utilized as cores in Ir‐based core–shell electrocatalysts. For instance, Shan et al. developed a core–shell Ru@IrO_*x*_ catalyst comprising a significantly lattice‐strained Ru core and a partially oxidized IrO_*x*_ shell.^[^ ^100^ ^]^ Using a sequential polyol method, Ru@IrO_*x*_ core–shell nanocrystals were synthesized in this investigation. In the synthesis process, a Ru precursor was reduced by ethylene glycol, and then an Ir precursor was added to form the IrO_*x*_ shell coating on the Ru cores. Characterization techniques such as X‐ray photoelectron spectroscopy (XPS) and X‐ray absorption spectroscopy (XAS) revealed that the interface between Ru and IrO_*x*_ caused in electron redistribution, leading to an increased valence state of the IrO_*x*_ shell. This charge redistribution at the Ru–IrO_*x*_ heterojunction optimized the adsorption of oxygen intermediates, thereby enhancing the OER activity. Due to the favorable valance state of active sites, the Ru@IrO_*x*_ nanostructure achieves an overpotential of only 282 mV, allowing it to deliver and anodic current density of 10 mA cm^−2^ (Figure 5E). Furthermore, a chronoamperometric test conducted at a potential of 1.55 V_RHE_ demonstrated the exceptional stability of Ru@IrO_*x*_, as it showed negligible activity loss even after 24 h of continuous operation. In contrast, RuIrO_*x*_ experienced a significant decline in activity, losing nearly 80% of its initial performance within less than 1 h (Figure 5F). Additionally, analysis using inductively coupled plasma mass spectrometry (ICP‐MS) revealed minimal dissolution of Ir and Ru from the Ru@IrO_*x*_ nanocrystal, further confirming its outstanding stability.

Audichon et al. fabricated a core–shell‐like structure of IrO_2_@RuO_2_, where IrO_2_ was coated on RuO_2_. This composite displayed improved OER activity and stability in comparison to pure RuO_2_ and IrO_2_.^[^ ^23^ ^]^ The authors suggested that the close interaction between RuO_2_ and IrO_2_ could facilitate a synergistic effect between the two oxides. However, it should be acknowledged that the size of particle IrO_2_@RuO_2_ was significantly smaller than that of pure RuO_2_ or IrO_2_, which might have contributed to the improved OER activity. Consequently, more substantial evidence is required to support the claim of an enhanced synergistic effect between RuO_2_ and IrO_2_ in the core–shell‐like IrO_2_@RuO_2_ structure. Huang et al. synthesized Ir–ZrTaO_*x*_ by using ethylene glycol reduction procedure to control the Ir nanoparticles onto a ZrO_*x*_/TaO_*x*_ mix‐supporting material.^[^ ^101^ ^]^ The Ir–ZrTaO_*x*_ catalyst maintains 1000 h electrolysis and displays two‐order higher mass activity than IrO_2_. It is to be noted that after 200 h operation in a PEM electrolyzer, 1/4 Ir loading shows low deterioration (4.7 μV h^−1^). Shi et al. found the unique Ir single site that was embedded in γ‐MnO_2_ and contained 0.87% Ir.^[^ ^102^ ^]^ The Ir–MnO_2_ shows a 218 mV of overpotential and rather outstanding TOF (7.7 s^−1^). In addition, there is no evidence of an increase in the rate of bulk cationic or anionic migration or structural reconstruction during OER. Ir concentration in electrolyte observed a small decrease (56 ppb) after 650 h stability run. Chau et al. decorated IrO_2_ clusters on reduced TiO_2_ (blue TiO_2_), showed higher OER activity than IrO_2_ due to the synergistic advantages of covalently bonded Ir with hydroxyl groups of blue TiO_2_ in acidic conditions.^[^ ^103^ ^]^ The Ir cluster was generated and deposited on the surface of blue TiO_2_ that led to increase in the active surface area and enhanced the current density. The IrO_2_ blue TiO_2_ catalyst exhibits an overpotential of 342 mV lower than IrO_2_, and the mass activity is 1.38 A mgIr^−1^, 27 times greater than that of IrO_2_.

### 3.4 Metal‐Doped Ir Oxide‐Based OER Catalyst

**Figure 6:**
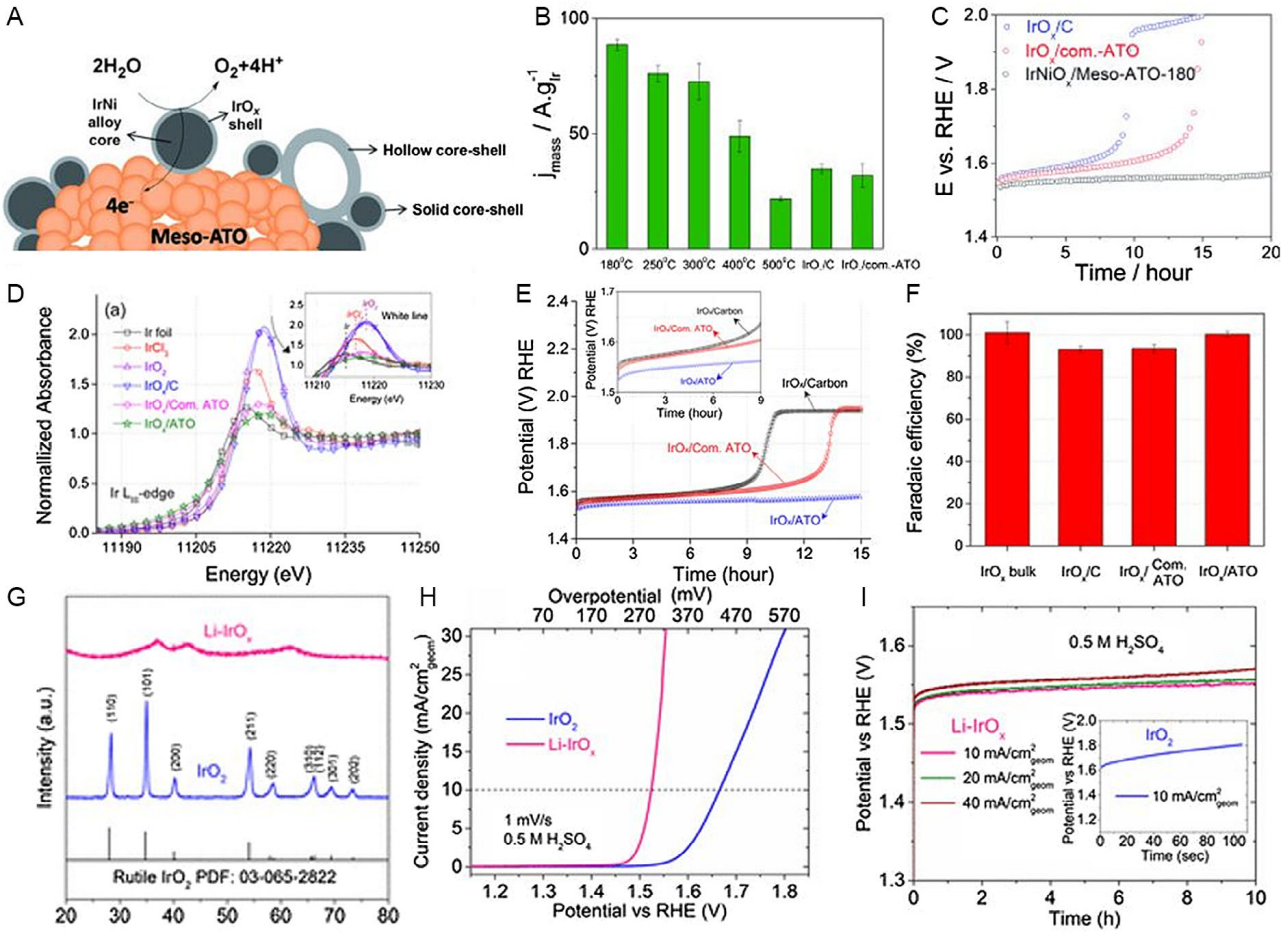
A) Scheme of OER on the IrO_*x*_ shell of IrNiO_*x*_ core–shell supported on Mes‐ATO. B) Ir mass‐based activity at overpoetential 280 mV of IrO_*x*_/C, IrO_*x*_/Com.‐ATO and IrNiO_*x*_/Meso‐ATO‐T. C) Chronopotentiometric stability measurements at 1 mA cm^−2^.^[^ ^104^ ^]^ (A,B,C) Reproduced with permission.^[^ ^97^ ^]^ Copyright 2015, Wiley. D) Normalized XANES spectra. E) Constant current stability test of IrO_*x*_/ATO electrocatalysts for OER in comparison to reference catalysts measured under identical conditions. F) Faradic efficiency of IrO_*x*_/ATO.^[^ ^105^ ^]^ (D,E,F) Reproduced with permission.^[^ ^98^ ^]^ Copyright 2016, American Chemical Society. G) XRD patterns of rutile IrO_2_ and amorphous Li–IrO_*x*_. H) LSV curves of rutile IrO_2_ and amorphous Li–IrO_*x*_ in 0.5 M H_2_SO_4_. I) Chronoamperometry test of amorphous Li–IrO_*x*_ at 10, 20, and 40 mA cmgeom^−2^; inset shows that of rutile IrO_2_ at 10 mA cmgeom^−2^.^[^ ^106^ ^]^ (G,H,I) Reproduced with permission.^[^ ^99^ ^]^ Copyright 2019, American Chemical Society.

In the pursuit of developing enhanced catalysts for acidic OER, a promising approach involves the artificial design of nanostructured materials with different composition, morphology, structure, and electronic environment. Nong et al. effectively produced IrNi@IrO_*x*_ OER catalyst core/shell nanoparticles through dealloying and selective oxidation of bimetallic nanoparticles. Remarkably, the structure of IrNi@IrO_*x*_ core/shell catalyst exhibited a 3 times improvement in catalytic activity compared to commercial catalysts, likely IrO_2_ and RuO_2_.^[^ ^63^ ^]^ Additionally, the researcher introduced a novel catalyst/support couple concept by investigating mesoporous ATO‐supported IrNi_*x*_@IrO_*x*_ core/shell nanoparticles for efficient OER (**Figure** **6**A). The catalyst with support demonstrated an oxygen evolution rate 2.5 times higher Ir‐mass‐based activity compared to commercial IrO_*x*_ (Figure 6B), and it maintained stability for 20 h at 1 mA cm^−2^ (Figure 6C).^[^ ^104^ ^]^ The exceptional OER activity was attributed to the kinetically frustrated oxidized Ir shell, while the mesoporous ATO support facilitated catalyst dispersion and provided superior corrosion resistance. Strasser et al.^[^ ^105^ ^]^ investigated on the impact of redox‐active metal–oxide supports on the redox stability of Ir (IrO_*x*_ nanoparticles on an ATO aerogel support). For comparison purposes, high surface area carbon was also employed as a support for IrO_*x*_. Through X‐ray absorption near edge structure (XANES) (Figure 6D) and XPS measurements, it was observed that the carbon‐supported oxidized Ir particles exhibited a redox state of +4 exclusively, while the Ir/IrO_*x*_/ATO system showed evidence of metal/metal–oxide support interactions (MMOSI). These interactions stabilized metal particles on ATO at sustained lower Ir oxidation states Ir^3.2+^, thereby inhibiting the growth of higher valent Ir oxide layers that degrade catalyst stability. Under constant current and potential, the electrochemical durability and charge‐transfer kinetics of the electrocatalysts were then evaluated. The analysis confirmed that the ATO support mitigates Ir^z+^ dissolution due to a more substantial MMOSI effect. The IrO_*x*_/ATO electrochemical stability and reference materials were evaluated by subjecting them to fixed water oxidation current densities for 15 h under strong acid (pH 1) (Figure 6E). The electrode potential for IrO_*x*_/C increased at 1.9 V_RHE_ after 9 h, and for IrO_*x*_/Com.‐ATO, it rose after 13 h and then leveled off. The overall voltage stability of the electrocatalysts followed the order: IrO_*x*_/C < IrO_*x*_/Com.‐ATO < IrO_*x*_/ATO. Both IrO_*x*_/ATO and bulk IrO_*x*_ exhibited a Faradaic efficiency of 100% for the OER (Figure 6F), indicating their high selectivity toward the water oxidation reaction. Ni dissolution during catalysis generated a distinct oxygen ligand environment for hole‐doped IrNi@IrO_*x*_ core/shell nanoparticles.^[^ ^39^ ^]^ It is to be observed that the distinctive catalyst exhibits shortened Ir—O metal–ligand bonds, and its Ir oxide shell contains an unusually high number of *d*‐band holes. It is observed that the distinctive catalyst has shortened Ir—O metal–ligand bonds and an unusually high number of *d*‐band pores in its Ir oxide shell. Notably, the Ni‐leached IrNi@IrO_*x*_ nanoparticles catalyst with a core/shell structure is 25 times more active than rutile IrO_2_ nanoparticles at an applied potential of 1.53 V_RHE_.^[^ ^39^ ^]^ Liu et al. reported the incorporating lithium ions into Ir oxide results in amorphous Ir oxide (Li–IrO_*x*_), showing remarkable water oxidation activity.^[^ ^106^ ^]^ The Li–IrO_*x*_ X‐ray diffraction (XRD) exhibits weak and broad peaks, contrasting with commercial IrO_2_, indicating its amorphous nature (Figure 6G). It achieved an outstanding OER current density of 10 mA cm^−2^ at an overpotential of 270 mV during 10 h of continuous operation in an acidic electrolyte (Figure 6H,I). In situ, XAS under OER operando conditions shows that amorphous Li–IrO_*x*_ undergoes oxidation to higher oxidation states and experiences shrinkage in the Ir—O bond, which is not observed in rutile IrO_2_. This suggests that the amorphous Li–IrO_*x*_ structure contains more “flexible” disordered [IrO_6_] octahedrons with higher oxidation states compared to the periodically interconnected “rigid” [IrO_6_] octahedrons in crystalline IrO_2_. These structural and oxidation state differences make amorphous Li–IrO_*x*_ more electrophilic, thereby enhancing the fast turnover of water oxidation.

Zaman et al. introduced tiny IrO_2_ anchored on 1D Co_3_O_4_ in an effort to develop efficiency and inexpensive OER electrocatalyst.^[^ ^107^ ^]^ The composition of Ir/Co (0.06/0.07) showed the exceptional performance, which is attributed to the synergistic effect of metal cations due to their electronic modulations as disclosed by XPS analysis and the experimentally confirmed significance of 1D nanorod‐shaped substrate particle assembly. Further Cheng et al. deposited IrO_*x*_ nanoparticles on the surface of Ti‐based nanorods to construct a 3D structure. The obtained catalyst exhibits remarkable in acidic OER (200 mV overpotential). In addition, no evident increase in potential was observed even after a 100 h chronopotentiometry test at 10 mA cm^−2^ and 700 cycles of cyclic voltammetry.^[^ ^108^ ^]^ In situ XAS measurements and DFT calculations supported their proposition that the mixed valence of Ir and the high level of OH concentration of the catalyst played vital roles in facilitating the acidic OER. Lebedev and Coperet introduced a method for immobilizing 1.5 nm Ir nanoparticles on a support of conductive indium tin oxide (ITO) film, which had been fabricated on a fluorine‐doped tin oxide (FTO) substrate.^[^ ^109^ ^]^ The electrode obtained from this process exhibited excellent activity and stability for acidic OER, achieving a current density of 10 A gIr^−1^ and an overpotential of 240 mV in a 0.1 M HClO_4_ electrolyte. During acidic OER conditions, it was observed that the interior of Ir nanoparticles maintained its metallic state, while a surface layer composed of amorphous Ir oxyhydroxide emerged, which played a crucial role in achieving the high OER performance. Wen et al. conducted a study where multiwalled carbon nanotubes (CNTs) were utilized as a support for ultrafine IrO_2_ particles to improve their performance in acidic OER conditions.^[^ ^110^ ^]^ The resulting composite, known as IrO_2_@CNT, contained 2.0 wt% IrO_2_ and exhibited remarkable OER activity. It required only 272 mV of overpotential to achieve a current density of 10 mA cm^−2^ in 0.5 M H_2_SO_4_ and demonstrated structural integrity even after continuous testing for 12 h at 10 mA cm^−2^. The synergistic interaction between IrO_2_ and CNT was proposed as the primary contributing factor for the enhanced performance of IrO_2_@CNT in acidic OER. In recent studies, other materials such as graphdiyne^[^ ^111^ ^]^ and B,N‐co‐doped reduced graphene oxide^[^ ^112^ ^]^ have also been investigated as supports for IrO_*x*_ to enhance its performance in acidic OER. The dissolution characteristics of Ir are strongly influenced by its coordination environment, which governs the interaction between Ir and water molecules. Modifying the coordination environment through doping offers a convenient approach to tune the dissolution behavior of Ir. Lee et al. conducted a study where they introduced Pt dopant into an Ir oxide‐based nanoframe. The presence of Pt dopant had a direct impact on the ratio between catalytically active Ir(III) species and stable Ir(IV) species, resulting in improved activity for the OER as well as enhanced durability of the catalyst.^[^ ^113^ ^]^

### 3.5 Pyrochlore Iridate Oxide‐Based OER Catalyst

Complex oxides known as pyrochlore oxides have garnered interest in the field of OER applications.^[^ ^114^, ^115^, ^116^ ^]^ Studies have focused on investigating Ir‐based pyrochlore oxides due to their demonstrated effectiveness and stability as OER electrocatalysts while also minimizing the reliance on precious metals.^[^ ^117^ ^]^ Pyrochlores, characterized by the general formula A_2_B_2_O_6_O′, belong to a class of complex oxides. The A‐site contains alkaline earth metals like Bi and Pb, while the B‐site has elements like Ir, Ru, Os, Ti, Nb, and Sn.^[^ ^118^ ^]^ Pyrochlore structures typically contain two types of oxygen anions: O anions act as bridges between both A and B cation sites (A–O–B) while the O′ anions exclusively connect the A cation sites (A–O′–A). This unique arrangement allows for the presence of flexible BO_6_ and A_2_O′ sublattice structures within pyrochlore oxides. Using different A and B cations, pyrochlores can easily change their properties due to their structural flexibility. The significant interaction between spin–orbital coupling and electron correlations has drawn attention to the 5*d* pyrochlore iridate oxides R_2_Ir_2_O_7_, where R is a rare‐earth metal ion.^[^ ^117^, ^119^ ^]^ This interaction accurately modulates R ions to achieve and fine‐tune interesting states like insulators, semimetals, and metals.^[^ ^120^, ^121^ ^]^ Such fine‐tuning provides a platform for exploring the catalytic performance based on electronic structure.

**Figure 7:**
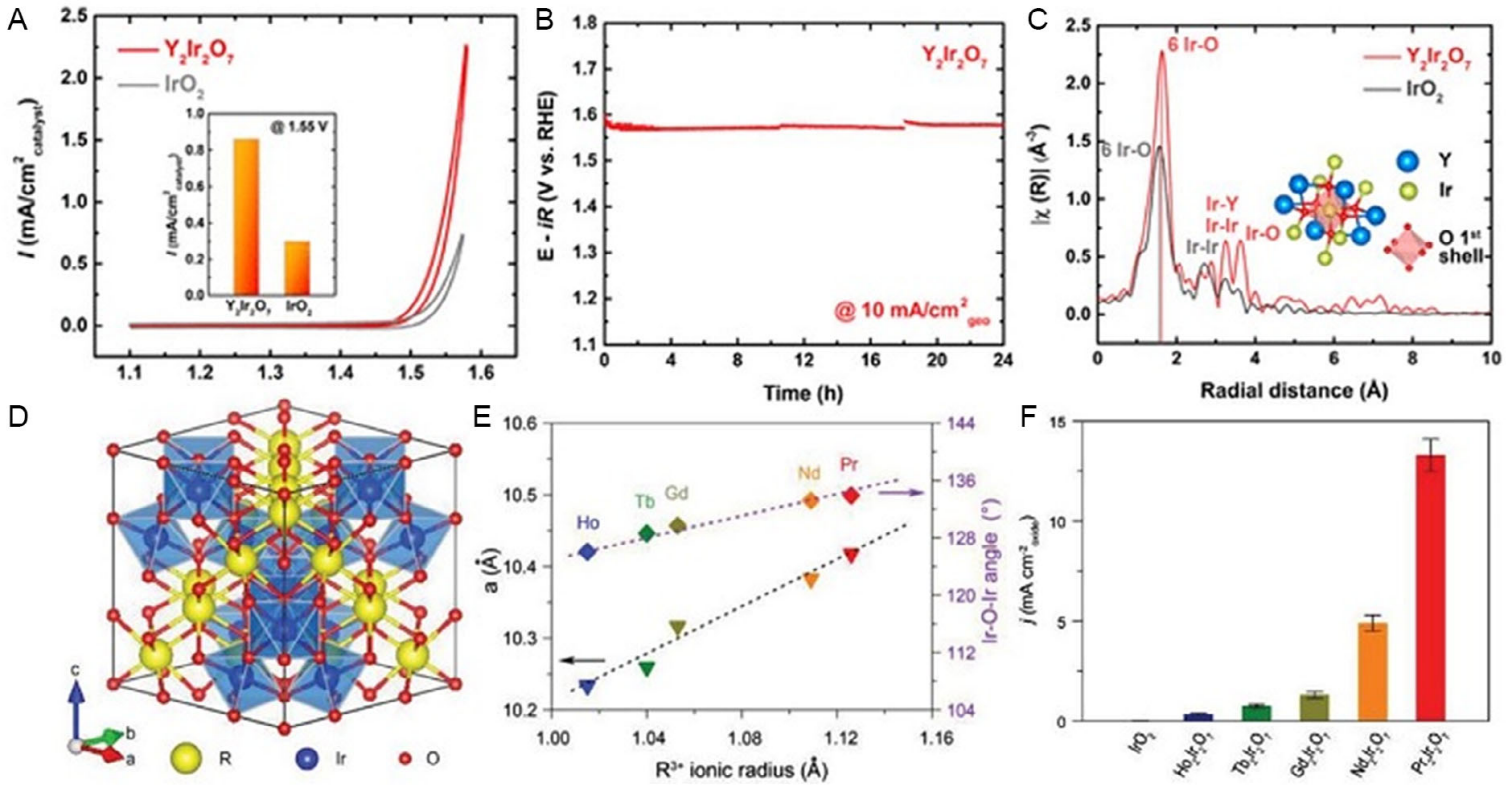
A) LSV curves of Y_2_Ir_2_O_7_. B) OER stability analysis of Y2Ir2O7 at 10 mA cm^−2^_geo._ C) EXAFS of Ir LIII‐edge analysis of Y_2_, Ir_2_O_7_, and IrO_2_, respectively. Inset illustrates the first shell oxygens in the unit.^[^ ^122^ ^]^ (A,B,C) Reproduced with permission.^[^ ^115^ ^]^ Copyright 2018, American Chemical Society. D) Crystal structure of R_2_Ir_2_O_7_. E) R^3+^ ionic radius‐dependent lattice constant *a* and Ir—O—Ir bond angle. F) Comparing current densities at an overpotential of 300 mV in 0.1 M HClO_4_.^[^ ^123^ ^]^ (D,E,F) Reproduced with permission.^[^ ^116^ ^]^ Copyright 2019, Wiley.

For instance, Yang and co‐workers synthesized a single‐phase pyrochlore‐type Y_2_Ir_2_O_7_ electrocatalyst using the sol–gel method. This catalyst exhibits remarkable activity and stability in promoting the OER in acidic conditions.^[^ ^122^ ^]^ At 320 mV overpotential, the Y_2_Ir_2_O_7_ electrocatalyst exhibited a specific current density of 0.86 mA cm^−2^_catalyst_, which was approximately 3 times greater than that of the reference catalyst IrO_2_ (0.30 mA cm^−2^_catalyst_) (**Figure** **7**A). Chronopotentiometry analysis demonstrated that the overpotential remained stable for 24 h at a 10 mA cm^−2^ current density (Figure 7B). Moreover, the catalyst showed excellent physical stability as no new shell formation was detected after the OER test. The XAS results were employed to investigate the single‐electron filling in the e″ orbital of Ir 5*d* states, attributed to strong spin–orbit coupling, which likely contributes to the improved OER activity (Figure 7C). Shang et al. synthesized R_2_Ir_2_O_7_ (R = Ho, Tb, Gd, Nd, and Pr) polycrystalline powders were produced using sol–gel and investigated the connection between catalyst acidic OER activity and electronic structures (Figure 7D).^[^ ^123^ ^]^ As the R ionic radius increases from Ho to Pr, the bond angle between Ir and oxygen expands, resulting in a chemical pressure effect and lattice expansion as demonstrated in Figure 7E. The acidic OER activity of R_2_Ir_2_O_7_ in 0.1 M HClO_4_ was found to be strongly dependent on the R ionic radius. Notably, the OER activity of R_2_Ir_2_O_7_ significantly increases with increasing R ionic radius. Remarkably, all the R_2_Ir_2_O_7_ oxides exhibit much higher OER activity than IrO_2_, and Pr_2_Ir_2_O_7_ demonstrates a mass activity approximately 21 times higher than that of IrO_2_. As R shifted from Ho to Pr, current density increased greatly. Meanwhile, onset potential dropped from 1.49 to 1.41 V; overpotential decreased from 500 to 290 mV. All pyrochlore iridates have higher mass activity than commercial IrO_2_ nanoparticles. Pr_2_Ir_2_O_7_ had the highest mass activity of 424.5 A g^−1^Ir at an overpotential of 300 mV, 21 times higher than commercial IrO_2_ nanoparticles (20.4 A g^−1^Ir). As the R ionic radius increases, resistivity measurements reveal that the pyrochlore R_2_Ir_2_O_7_ oxides suffer a transition from insulator to semimetal to metal. This transition is attributed to the weakening of electron correlations as the R ionic radius increases, as a result of the broadening of Ir 5d bandwidths caused by the increase in R ionic radius, which reduces the electron correlations. This results in a transition from insulator to metal and a stronger hybridization between Ir 5*d* and O 2*p* orbitals. The enhanced conductivity and covalency of Ir—O bonds contribute to the superior OER activity observed in Nd_2_Ir_2_O_7_ and Pr_2_Ir_2_O_7_. Sun et al. conducted a study to investigate the correlation between the IrO_6_ coordination geometry in the oxide structure and the OER performance of IrO_2_, Bi_2_Ir_2_O_7_, and Pb_2_Ir_2_O_6.5_.^[^ ^124^ ^]^ It was observed that the IrO_6_ octahedron exhibits varying levels of distortion, with Bi_2_Ir_2_O_7_ < IrO_2_ < Pb_2_Ir_2_O_6.5_. The OER activity in 0.1 m HClO_4_ was found to be strongly influenced by the degree of IrO_6_ distortion, following the activity order of Pb_2_Ir_2_O_6.5_ > IrO_2_ > Bi_2_Ir_2_O_7_. DFT calculations revealed that Pb–Ir has a broader bandwidth compared to IrO_2_, promoting the overlap of O 2*p* (intermediate adsorbate) with the valence band, resulting in enhanced OER activity. The transition metal‐doped IrO_2_ (Cu, Co, and Ni) has shown that OER activity enhancements are linked to defects and lattice disorders caused by crystal structure mismatch. In conclusion, the distorted IrO_6_ geometry plays a significant role in its OER activity. Based on these findings, altering IrO_6_ octahedral geometry through doping or using structures with distorted coordination can enhance OER activity.

### 3.6 Ir‐Based Perovskite Oxides as OER Catalyst

In addition to alloying with nonprecious metals/metal oxides, an alternative and prospective method to reduce the reliance on noble metals in acidic OER electrocatalysts is important. In this regard, the designing and synthesizing the complex perovskite oxides based on Ir or Ru considered as encouraging strategy for enhanced OER activity in acid media. Perovskites, with the general formula ABO_3_, have been extensively studied for various applications. Typically, the A‐site can be composed of alkaline‐earth metals (such as Ca, Sr, or Ba), rare‐earth metals (such as La or Pr), or a combination of the two. In contrast, a transition metal ion occupies the B‐site, forming a coordinated structure with sixfold octahedra (BO_6_) composed of oxygen ions and oxygen atoms.^[^ ^125^ ^]^ Extensive research has demonstrated that by adjusting the valence states and the number of atoms present at the A‐ and B‐sites, it becomes possible to control the lattice oxygen content and electronic structure. Consequently, the introduction of oxygen defects can be achieved, leading to a significant impact on the catalytic activity of the material toward the OER.^[^ ^8^ ^]^ While the majority of application‐driven research on perovskite materials in OER electrocatalysis has concentrated on alkaline media, efforts have been made to investigate the relationship between structure and performance. This has resulted in the development of catalysts with triple perovskite structures^[^ ^126^ ^]^ and antiperovskite structures.^[^ ^127^ ^]^ In recent years, there has also been extensive investigation into the catalytic performance of Ir‐based perovskites under acidic conditions. The above aspect will be the main focus of the subsequent discussion.

Jaramillo et al. demonstrated for the first time the suitability of AIrO_3_ and known as a single perovskite oxide, was explored for its applicability in acidic water oxidation reactions.^[^ ^128^ ^]^ The researchers prepared a pseudocubic SrIrO_3_ thin film, with thicknesses of either 60 or 100 nm, through pulsed laser deposition onto a SrTiO_3_ substrate. They conducted chronopotentiometry tests at a current density of 10 mA cm^−2^ in a 0.5 M H_2_SO_4_ solution and observed an enhancement in the OER activity of SrTiO_3_ during the initial 2 h period. Subsequently, a 30 h test revealed that SrTiO_3_ displayed an overpotential range of 270–290 mV.

**Figure 8:**
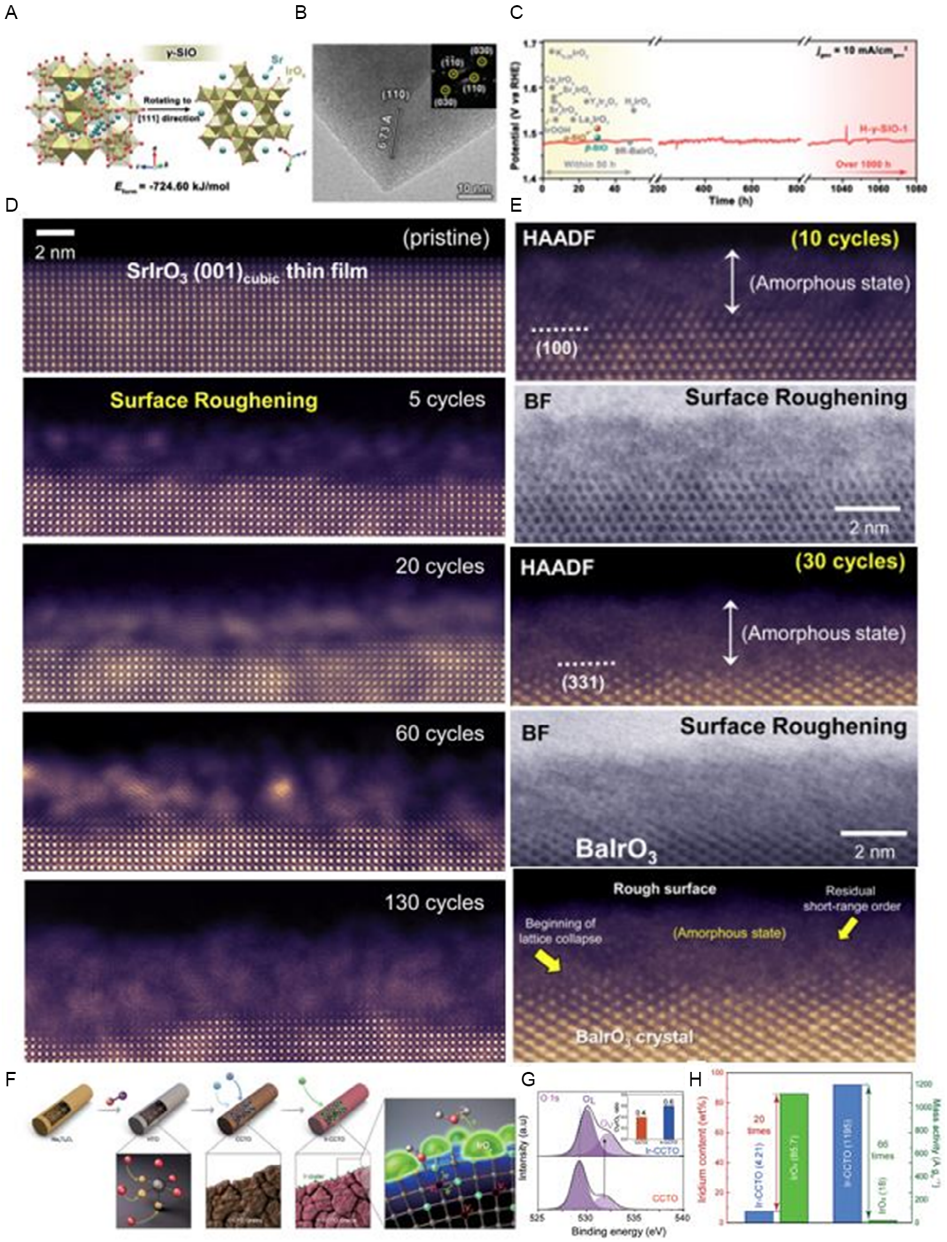
A) Crystal structure of γ‐SIO. B) High‐resolution transmission electron microscopy (HR‐TEM) images of H‐γ‐SIO. C) Chronopotentiometric curves for OER.^[^ ^129^ ^]^ (A,B,C) Reproduced with permission.^[^ ^122^ ^]^ Copyright 2023, Wiley. D) HAADF images of pseudocubic SrIrO_3_. E) Monoclinic BaSrIrO_3_ after CV in 0.5 M H_2_SO_4_ solution.^[^ ^130^ ^]^ (D,E) Reproduced with permission.^[^ ^123^ ^]^ Copyright 2019, Elsevier. F) Schematic illustration for the incorporation of Ir dopant into the CCTO NBs. F) XPS spectra of O 1*s*. G) Ir mass activity of Ir‐CCTO and IrO_2_.^[^ ^131^ ^]^ (F,G) Reproduced with permission.^[^ ^124^ ^]^ Copyright 2023, Wiley.

Zou et al.^[^ ^129^ ^]^ presented the phase‐selective synthesis of metastable strontium iridates with an open‐framework structure (γ‐SIO), leading to an unexpectedly highly active and stable oxygen evolution nanoelectrocatalyst. **Figure** **8**A,B shows the Sr^2+^/H^+^ ion exchange in an acidic environment and the in situ structural rearrangement under electrocatalytic conditions. Compare to the perovskite‐structured iridates (SrIrO_3_), the open‐framework iridates undergo rapid proton exchange in acid without undergoing framework amorphization and reform into ultrasmall, surface‐hydroxylated rutile nanocatalysts arranged along the (200) crystal plane. These unique microstructural characteristics benefit the oxidation of hydroxyls and the formation of O—O bonds in the electrocatalytic cycle. As a result, the nanocatalyst derived from open‐framework iridates demonstrates comparable catalytic activity to the most active Ir‐based oxygen evolution electrocatalysts in acidic conditions, and it retains its catalytic activity for over 1000 h (Figure 8C).

Chung et al. employed atomic‐resolution STEM to visualize the surface reconstruction of AIrO_3_ perovskite oxides, where A represents Sr or Ba.^[^ ^130^ ^]^ Three types of AIrO_3_ perovskite oxides were prepared: pseudocubic SrIrO_3_ thin films deposited on (LaAlO_3_)_0.3_(SrAl_0.5_Ta_0.5_O_3_)_0.7_ substrate using reactive‐oxide molecular‐beam epitaxy, monoclinic SrIrO_3_, and BaIrO_3_ synthesized through a conventional solid‐state reaction. Analysis of HAADF images revealed that all three AIrO_3_ perovskite oxides exhibited surface roughening and structural changes due to the leaching of Sr or Ba from the A site when subjected to anodic current (Figure 8D). Rapid leaching of Sr during the initial OER test formed rutile IrO_2_ nanocrystallites on pseudocubic SrIrO_3_ thin films, which later turned amorphous (Figure 8D). Conversely, amorphous IrO_*x*_ was observed on the surfaces of monoclinic SrIrO_3_ and BaIrO_3_ after the OER test (Figure 8E). Jaramillo and co‐workers found that the creation of IrO_*x*_ groups increased the active surface area and boosted AIrO_3_ extrinsic OER activity through cycling.^[^ ^128^ ^]^ During electrochemical testing in acidic conditions, Sr leaching created mosaic‐shaped Sr‐deficient IrO_*x*_ on the surface of other pseudocubic SrIrO_3_ thin films, confirming OER activity. Continuous production of IrO_*x*_ active species is key to electrocatalytic OER activity. In contrast, the intrinsic OER activity of AIrO_3_, normalized to the electrochemically active surface area (ECSA), decreased. Cherevko et al. conducted a study on the electrocatalytic activity of A_2_BIrO_6_ double perovskite oxides based on Ir (A = Ba, Sr; B = Pr, Nd, and Y) for acidic water oxidation.^[^ ^45^ ^]^ The A_2_BIrO_6_ compounds, like Ba_2_BIrO_6_, experienced cation leaching in a 0.1 M HClO_4_ solution at open‐circuit voltage, resulting in higher Ir dissolution (30–40 wt%). For Ba_2_PrIrO_6_, prolonged immersion in the acidic solution caused complete leaching of both Ba and Pr ions, forming an amorphous IrO_*x*_ phase. This process dissolved isolated IrO_*x*_ octahedra, leading to the formation of activated oxygen with undercoordinated oxygen atoms. The amorphous IrO_*x*_ with activated oxygen significantly enhanced the OER activity by creating oxygen vacancy sites, replenished through water molecules or bulk oxygen adsorption or diffusion. However, oxygen vacancies also increased the rate of Ir dissolution in acidic solutions. Han et al.^[^ ^131^ ^]^ introduced Ir into Ca_3_Cu_4_O_12_ (CCTO) nanobelt perovskite structure by using hydrothermal and ion exchange method (Figure 8F). Ir is a substitutional dopant for Ti sites and an atomic seed for Ir‐nanocluster production on CCTO. Ir substitutional doping increases oxygen vacancies in the CCTO lattice due to its structural and electrical flexibility (Figure 8D). The presence of Ir increases M–O covalency, reducing charge‐transfer energy and increasing OER activity. Thus, Ir‐CCTO has ultrahigh mass activity 66 times higher than the benchmark IrO_2_ catalyst and exceptional catalytic activity and durability. The use of large dielectric materials in acidic OER has led to a breakthrough in mass activity, reaching up to 1000 A gIr^−1^. **Table** **2** shows recently developed Ir‐based efficient OER catalysts and their activity under acidic media.

**Table 2:** Ir‐based catalysts for enhanced OER activity and stability under acidic electrolyte

Catalyst	Electrolyte	Overpotential [mV] at 10 cm^−2^	Tafel slope [mV dec^−1^]	Stability	References
Ir nanotube	0.1 M HClO_4_	245	49.02	4 h at 10 mA cm^−2^	[133]
3D Ir superstructure	0.5 M H_2_SO_4_	270	40.8	8 h at 10 mA cm^−2^	[70]
Ir wavy nanowires	0.5 M HClO_4_	270	30.1	25 000 s at 10 mA cm^−2^	[134]
Mesoporous Ir nanosheets	0.5 M H_2_SO_4_	240	49	8 h at 10 mA cm^−2^	[135]
Amorphous Ir nanosheet	0.1 M HClO_4_	255	40	8 h at *ŋ* = 255 mV	[136]
Ir nanorod	0.5 M H_2_SO_4_	290	72.4	1000 cycles	[137]
RuIr nanocrystals	0.1 M HClO_4_	344	111.5	Decay continuously	[138]
Co–RuIr nanocrystals	0.1 M HClO_4_	235	66.9	25 h at 10 mA cm^−2^	[138]
IrNi nanocluster	0.1 M HClO_4_	Ŋ_9.1 _= 270	–	2 h at 5 mA cm^−2^	[80]
IrW nanodendrites	0.1 M HClO_4_	Ŋ_8.1 _= 300	56.6	3000 cycles	[85]
Ir_44_Pd_10_ nanocage	0.1 M HClO_4_	226	53.9	15 h at 10 mA cm^−2^	[82]
IrCoNi hollow nanocrystal	0.1 M HClO_4_	303	53.8	200 min at 5 mA cm^−2^	[84]
IrNiCu DNF	0.1 M HClO_4_	300	48	2500 cycles	[78]
RhIr nanoparticle	0.5 M H_2_SO_4_	292	101	8 h at 1.53 V_RHE_	[139]
Co–IrCu ONC/C	0.1 M HClO_4_	290	50	2000 cycles	[140]
Al–Ni–Co–Ir–Mo alloy	0.5 M H_2_SO_4_	275	55.2	48	[141]
IrCoNi PHNCs	0.1 M HClO_4_	303	53.8	–	[84]
P–IrCu_1.4_	0.05 M H_2_SO_4_	311	53.9	10 h at 10 mA cm^−2^	[142]
4H/fcc Au@Ir nanoribbon	0.5 M H_2_SO_4_	296	51.4	–	[83]
Amorphous IrO_*x*_	1 M H_2_SO_4_	220	34	24 h at 1 mA cm^−2^	[89]
IrO_2_ nanoneedle	1 M H_2_SO_4_	313	57	2	[91]
RuIrO_*x*_ nano‐netcages	0.5 M H_2_SO_4_	233	42	3000 cycles	[143]
Ru@IrO_*x*_ core/shell	0.05 M H_2_SO_4_	282	69.1	24 h at 1.55 V_RHE_	[100]
Ir_0.6_Cr_0.4_O_*x*_ nanowires	0.5 M H_2_SO_4_	250	69	25 h at 10 mA cm^−2^	[144]
Single layer IrOOH nanosheets	0.1 M HClO_4_	344	58	14 h at 10 mA cm^−2^	[145]
IrNi@IrO_*x*_	0.05 M H_2_SO_4_	300	–	2500 cycles	[39]
IrCo@IrO_*x*_‐3L NDs	0.05 M H_2_SO_4_	247	49	10 h at 2.5 mA cm^−2^	[146]
Pd@Ir3L	0.1 M HClO_4_	263	56.3	2000 cycles	[147]
Ni_0.34_Co_0.46_Ir_0.2_O_φ_	0.1 M HClO_4_	280	–	20 000 s at 10 mA cm^−2^	[148]
6H SrIrO_3_	0.5 M H_2_SO_4_	248	–	30 h at 10 mA cm^−2^	[149]
Co‐doped 6H‐SrIrO_3_	0.1 M HClO_4_	235	51.8	20 h at 10 mA cm^−2^	[150]
Ir‐STO	0.1 M HClO_4_	247	–	20 h at 10 mA cm^−2^	[151]
Ir‐SA@Fe@NCNT	0.5 M H_2_SO_4_	250	58.2	11.5 h at 10 mA cm^−2^	[73]
Ir‐CCTO	0.1 M HClO_4_	280	44.7	20 h at 20 mA cm^−2^	[131]
Ir‐HTO	0.1 M HClO_4_	266	41	50 h at 10 mA cm^−2^	[152]
Y_2_Ir_2_O_7_	0.1 M HClO_4_	–	51.8	24 h at 10 mA cm^−2^	[122]
Pr_2_Ir_2_O_7_	0.1 M HClO_4_	290	–	10 000 s at 10 mA cm^−2^	[123]

## 4 Conclusions and Perspectives

In conclusion, the search for affordable, highly efficient, and stable catalyst in acid media for the OER is crucial in the advancement of energy generation, conversion, and storage technologies, such as electrolyzers, fuel cells, regenerative fuel cells, metal–air batteries, etc. to achieve a clean energy in future. In this review, we have examined the mechanism of the acidic OER for PEMWEs, combining DFT simulations with in situ/operando characterization techniques. Moreover, the catalysts follow LOM found to be comparatively higher OER performance than AEM due to Ir species dissolution rapidly. Furthermore, we have provided an overview of recent advancements in the development of various Ir‐based catalysts for the acidic OER, including noble‐metal nanomaterials, alloys, precious‐metal‐based oxides, pyrochlores, perovskites, single‐atom materials, and nonprecious‐based catalysts, respectively. Moving forward, future research and development efforts in the field of acidic OER electrocatalysis should focus on the design and fabrication of advanced materials, considering the following aspects: 1) It is important to develop intrinsically stable noble metal (Ir/Ru) free catalysts that consist of high resistance to corrosion under acidic OER condition. So that cost‐effective catalysts with enhanced activity as well as stability can be achieved in PEMWEs. 2) A strategy needs to explore the researcher as future aspect on doping Ir/Ru at different exposed facets of metal oxides and/or carbon‐based catalysts with higher degree of graphitization to synthesize structurally stable catalyst for efficient OER with extraordinary stability. 3) Researchers need to explore various synthesis methods such as electrodeposition, microwave‐assisted methods, etc. to synthesize thermodynamical stable catalyst for acidic OER activity in future. 4) The Ir‐based acidic OER catalyst follows LOM as reported extensively; however, most of the relevant theoretical studies still follow AEM. Thus, it is important to understand the LOM role in acidic OER performance as well as stability for Ir‐based catalyst in future studies.

## Conflict of Interest

The authors declare no conflict of interest.

## References

[1] J. A. Turner , Science 2004, 305, 972. 15310892 10.1126/science.1103197

[2] S. Chu , A. Majumdar , Nature 2012, 488, 294. 22895334 10.1038/nature11475

[3] P. Moriarty , D. Honnery , Int. J. Hydrogen Energy 2007, 32, 1616.

[4] S. Anantharaj , V. Aravindan , Adv. Mater. 2020, 10, 1902666.

[5] C. Wei , R. R. Rao , J. Peng , B. Huang , I. E. L. Stephens , M. Risch , Z. J. Xu , Y. Shao-Horn , Adv. Mater. 2019, 31, 1806296. 10.1002/adma.201806296 30656754

[6] Z. J. Xu , X. Wang , Chem. – A Eur. J. 2020, 26, 3897. 10.1002/chem.202000909 32167201

[7] Z. W. Seh , J. Kibsgaard , C. F. Dickens , I. Chorkendorff , J. K. Nørskov , T. F. Jaramillo , Science 2017, 355, eaad4998. 28082532 10.1126/science.aad4998

[8] J. Hwang , R. R. Rao , L. Giordano , Y. Katayama , Y. Yu , Y. Shao-Horn , Science 2017, 358, 751. 29123062 10.1126/science.aam7092

[9] H. Dau , C. Limberg , T. Reier , M. Risch , S. Roggan , P. Strasser , ChemCatChem 2010, 2, 724.

[10] S. Trasatti , Electrochim. Acta 1984, 29, 1503.

[11] M. Carmo , D. L. Fritz , J. Mergel , D. Stolten , Int. J. Hydrogen Energy 2013, 38, 4901.

[12] J. D. Holladay , J. Hu , D. L. King , Y. Wang , Catal. Today 2009, 139, 244.

[13] Q. Shi , C. Zhu , D. Du , Y. Lin , Chem. Soc. Rev. 2019, 48, 3181. 31112142 10.1039/c8cs00671g

[14] T. Reier , H. N. Nong , D. Teschner , R. Schlögl , P. Strasser , Adv. Mater. 2017, 7, 1601275.

[15] S. Cherevko , A. R. Zeradjanin , A. A. Topalov , N. Kulyk , I. Katsounaros , K. J. J. Mayrhofer , ChemCatChem 2014, 6, 2219.

[16] I. C. Man , H.-Y. Su , F. Calle-Vallejo , H. A. Hansen , J. I. Martínez , N. G. Inoglu , J. Kitchin , T. F. Jaramillo , J. K. Nørskov , J. Rossmeisl , ChemCatChem 2011, 3, 1159.

[17] J. Rossmeisl , Z.-W. Qu , H. Zhu , G.-J. Kroes , J. K. Nørskov , J. Electroanal. Chem. 2007, 607, 83.

[18] N. Danilovic , R. Subbaraman , K.-C. Chang , S. H. Chang , Y. J. Kang , J. Snyder , A. P. Paulikas , D. Strmcnik , Y.-T. Kim , D. Myers , V. R. Stamenkovic , N. M. Markovic , J. Phys. Chem. Lett. 2014, 5, 2474. 26277818 10.1021/jz501061n

[19] A. F. Pedersen , M. Escudero-Escribano , B. Sebok , A. Bodin , E. Paoli , R. Frydendal , D. Friebel , I. E. L. Stephens , J. Rossmeisl , I. Chorkendorff , A. Nilsson , J. Phys. Chem. B 2018, 122, 878. 28980810 10.1021/acs.jpcb.7b06982

[20] M. Escudero-Escribano , A. F. Pedersen , E. A. Paoli , R. Frydendal , D. Friebel , P. Malacrida , J. Rossmeisl , I. E. L. Stephens , I. Chorkendorff , J. Phys. Chem. B 2018, 122, 947. 29045788 10.1021/acs.jpcb.7b07047

[21] R. Kötz , S. Stucki , Electrochim. Acta 1986, 31, 1311.

[22] T. I. Valdez , K. J. Billings , J. Sakamoto , F. Mansfeld , S. R. Narayanan , ECS Trans. 2009, 25, 1371.

[23] T. Audichon , T. W. Napporn , C. Canaff , C. Morais , C. Comminges , K. B. Kokoh , J. Phys. Chem. C 2016, 120, 2562.

[24] S. Cherevko , S. Geiger , O. Kasian , N. Kulyk , J.-P. Grote , A. Savan , B. R. Shrestha , S. Merzlikin , B. Breitbach , A. Ludwig , K. J. J. Mayrhofer , Catal. Today 2016, 262, 170.

[25] M. V. ten Kortenaar , J. F. Vente , D. J. W. Ijdo , S. Müller , R. Kötz , J. Power Sources 1995, 56, 51.

[26] R. Frydendal , E. A. Paoli , B. P. Knudsen , B. Wickman , P. Malacrida , I. E. L. Stephens , I. Chorkendorff , ChemElectroChem 2014, 1, 2075.

[27] C. Spöri , J. T. H. Kwan , A. Bonakdarpour , D. P. Wilkinson , P. Strasser , Angew. Chem., Int. Ed. 2017, 56, 5994. 10.1002/anie.201608601 27805788

[28] L. She , G. Zhao , T. Ma , J. Chen , W. Sun , H. Pan , Adv. Funct. Mater. 2022, 32, 2108465.

[29] J. Fan , M. Chen , Z. Zhao , Z. Zhang , S. Ye , S. Xu , H. Wang , H. Li , Nat. Energy 2021, 6, 475.

[30] B. G. Pollet , Catalysts 2019, 9, 246.

[31] X. Zou , Y. Zhang , Chem. Soc. Rev. 2015, 44, 5148. 25886650 10.1039/c4cs00448e

[32] P. Shirvanian , F. van Berkel , Electrochem. Commun. 2020, 114, 106704.

[33] P. Millet , R. Ngameni , S. A. Grigoriev , N. Mbemba , F. Brisset , A. Ranjbari , C. Etiévant , Int. J. Hydrogen Energy 2010, 35, 5043.

[34] Y. Sun , J. Wu , Y. Xie , X. Wang , K. Ma , Z. Tian , Z. Zhang , Q. Liao , W. Zheng , Z. Kang , Y. Zhang , Adv. Funct. Mater. 2022, 32, 2207116.

[35] D.-Y. Kuo , H. Paik , J. Kloppenburg , B. Faeth , K. M. Shen , D. G. Schlom , G. Hautier , J. Suntivich , J. Am. Chem. Soc. 2018, 140, 17597. 30463402 10.1021/jacs.8b09657

[36] M. Peng , J. Huang , Y. Zhu , H. Zhou , Z. Hu , Y.-K. Liao , Y.-H. Lai , C.-T. Chen , Y.-H. Chu , K. H. L. Zhang , X. Fu , F. Zuo , J. Li , Y. Sun , ACS Sustain. Chem. Eng. 2021, 9, 4262.

[37] H. Wu , Y. Wang , Z. Shi , X. Wang , J. Yang , M. Xiao , J. Ge , W. Xing , C. Liu , J. Mater. Chem. A 2022, 10, 13170.

[38] Q. Dang , H. Lin , Z. Fan , L. Ma , Q. Shao , Y. Ji , F. Zheng , S. Geng , S.-Z. Yang , N. Kong , W. Zhu , Y. Li , F. Liao , X. Huang , M. Shao , Nat. Commun. 2021, 12, 6007. 34650084 10.1038/s41467-021-26336-2 PMC8516950

[39] H. N. Nong , T. Reier , H.-S. Oh , M. Gliech , P. Paciok , T. H. T. Vu , D. Teschner , M. Heggen , V. Petkov , R. Schlögl , T. Jones , P. Strasser , Nat. Catal. 2018, 1, 841.

[40] Z. Shi , X. Wang , J. Ge , C. Liu , W. Xing , Nanoscale 2020, 12, 13249. 32568352 10.1039/d0nr02410d

[41] J. Shan , Y. Zheng , B. Shi , K. Davey , S.-Z. Qiao , ACS Energy Lett. 2019, 4, 2719.

[42] T. Binninger , R. Mohamed , K. Waltar , E. Fabbri , P. Levecque , R. Kötz , T. J. Schmidt , Sci. Rep. 2015, 5, 12167. 26178185 10.1038/srep12167 PMC4503990

[43] J. H. Montoya , L. C. Seitz , P. Chakthranont , A. Vojvodic , T. F. Jaramillo , J. K. Nørskov , Nat. Mater. 2017, 16, 70. 10.1038/nmat4778 27994241

[44] A. Zagalskaya , V. Alexandrov , ACS Catal. 2020, 10, 3650.

[45] S. Geiger , O. Kasian , M. Ledendecker , E. Pizzutilo , A. M. Mingers , W. T. Fu , O. Diaz-Morales , Z. Li , T. Oellers , L. Fruchter , A. Ludwig , K. J. J. Mayrhofer , M. T. M. Koper , S. Cherevko , Nat. Catal. 2018, 1, 508.

[46] A. Grimaud , A. Demortière , M. Saubanère , W. Dachraoui , M. Duchamp , M.-L. Doublet , J.-M. Tarascon , Nat. Energy 2016, 2, article no. 16189.

[47] Z. Chen , X. Duan , W. Wei , S. Wang , B.-J. Ni , Nano Energy 2020, 78, 105392.

[48] N. Danilovic , R. Subbaraman , K. C. Chang , S. H. Chang , Y. Kang , J. Snyder , A. P. Paulikas , D. Strmcnik , Y. T. Kim , D. Myers , V. R. Stamenkovic , N. M. Markovic , Angew. Chem., Int. Ed. 2014, 53, 14016. 10.1002/anie.201406455 25297010

[49] A. R. Zeradjanin , A. A. Topalov , Q. V. Overmeere , S. Cherevko , X. Chen , E. Ventosa , W. Schuhmann , K. J. J. Mayrhofer , RSC Adv. 2014, 4, 9579.

[50] C. Roy , R. R. Rao , K. A. Stoerzinger , J. Hwang , J. Rossmeisl , I. Chorkendorff , Y. Shao-Horn , I. E. L. Stephens , ACS Energy Lett. 2018, 3, 2045.

[51] S. W. Lee , C. Baik , C. Pak , Catal. Today 2020, 358, 203.

[52] S. Lee , Y.-J. Lee , G. Lee , A. Soon , Nat. Commun. 2022, 13, 3171. 35676247 10.1038/s41467-022-30838-y PMC9177587

[53] A. Minguzzi , O. Lugaresi , E. Achilli , C. Locatelli , A. Vertova , P. Ghigna , S. Rondinini , Chem. Sci. 2014, 5, 3591.

[54] V. Pfeifer , T. E. Jones , J. J. V. Vélez , C. Massué , M. T. Greiner , R. Arrigo , D. Teschner , F. Girgsdies , M. Scherzer , J. Allan , M. Hashagen , G. Weinberg , S. Piccinin , M. Hävecker , A. Knop-Gericke , R. Schlögl , Phys. Chem. Chem. Phys. 2016, 18, 2292. 26700139 10.1039/c5cp06997a

[55] E. Willinger , C. Massué , R. Schlögl , M. G. Willinger , J. Am. Chem. Soc. 2017, 139, 12093. 28793758 10.1021/jacs.7b07079

[56] O. Kasian , S. Geiger , T. Li , J.-P. Grote , K. Schweinar , S. Zhang , C. Scheu , D. Raabe , S. Cherevko , B. Gault , K. J. J. Mayrhofer , Energy Environ. Sci. 2019, 12, 3548.

[57] R. V. Mom , L. J. Falling , O. Kasian , G. Algara-Siller , D. Teschner , R. H. Crabtree , A. Knop-Gericke , K. J. J. Mayrhofer , J.-J. Velasco-Vélez , T. E. Jones , ACS Catal. 2022, 12, 5174.

[58] V. Pfeifer , T. E. Jones , J. J. V. Vélez , R. Arrigo , S. Piccinin , M. Hävecker , A. Knop-Gericke , R. Schlögl , Chem. Sci. 2017, 8, 2143. 28507666 10.1039/c6sc04622c PMC5407268

[59] O. Kasian , J.-P. Grote , S. Geiger , S. Cherevko , K. J. J. Mayrhofer , Angew. Chem., Int. Ed. 2018, 57, 2488. 10.1002/anie.201709652 PMC5838529 29219237

[60] M. Scohy , S. Abbou , V. Martin , B. Gilles , E. Sibert , L. Dubau , F. Maillard , ACS Catal. 2019, 9, 9859.

[61] A. BalaKrishnan , N. Blanc , U. Hagemann , P. Gemagami , K. Wonner , K. Tschulik , T. Li , Angew. Chem., Int. Ed. 2021, 60, 21396. 10.1002/anie.202106790 PMC8518547 34343398

[62] T. Reier , M. Oezaslan , P. Strasser , ACS Catal. 2012, 2, 1765.

[63] H. N. Nong , L. Gan , E. Willinger , D. Teschner , P. Strasser , Chem. Sci. 2014, 5, 2955.

[64] P. Lettenmeier , J. Majchel , L. Wang , A. S. Gago , K. A. Friedrich , ECS Trans. 2016, 72, 1.

[65] J. E. Ferrer , L. L. Victori , Electrochim. Acta 1994, 39, 667.

[66] S. M. Alia , S. Pylypenko , K. C. Neyerlin , S. S. Kocha , B. S. Pivovar , ECS Trans. 2015, 69, 883.

[67] J. Zhang , G. Wang , Z. Liao , P. Zhang , F. Wang , X. Zhuang , E. Zschech , X. Feng , Nano Energy 2017, 40, 27.

[68] M. Ledendecker , S. Geiger , K. Hengge , J. Lim , S. Cherevko , A. M. Mingers , D. Göhl , G. V. Fortunato , D. Jalalpoor , F. Schüth , C. Scheu , K. J. J. Mayrhofer , Nano Res. 2019, 12, 2275.

[69] S. Cherevko , S. Geiger , O. Kasian , A. Mingers , K. J. J. Mayrhofer , J. Electroanal. Chem. 2016, 773, 69.

[70] Y. Pi , N. Zhang , S. Guo , J. Guo , X. Huang , Nano Lett. 2016, 16, 4424. 27249544 10.1021/acs.nanolett.6b01554

[71] B. M. Tackett , W. Sheng , S. Kattel , S. Yao , B. Yan , K. A. Kuttiyiel , Q. Wu , J. G. Chen , ACS Catal. 2018, 8, 2615.

[72] B. Jiang , T. Wang , Y. Cheng , F. Liao , K. Wu , M. Shao , ACS Appl. Mater. Interfaces 2018, 10, 39161. 30338972 10.1021/acsami.8b11970

[73] F. Luo , H. Hu , X. Zhao , Z. Yang , Q. Zhang , J. Xu , T. Kaneko , Y. Yoshida , C. Zhu , W. Cai , Nano Lett. 2020, 20, 2120. 32019309 10.1021/acs.nanolett.0c00127

[74] H.-S. Oh , H. N. Nong , T. Reier , M. Gliech , P. Strasser , Chem. Sci. 2015, 6, 3321. 28706696 10.1039/c5sc00518c PMC5490338

[75] A. Macchioni , Eur. J. Inorg. Chem. 2019, 2019, 7.

[76] L. Fagiolari , F. Zaccaria , F. Costantino , R. Vivani , C. K. Mavrokefalos , G. R. Patzke , A. Macchioni , Dalton Trans. 2020, 49, 2468. 31993601 10.1039/c9dt04306c

[77] L. Fagiolari , M. Bini , F. Costantino , G. Gatto , A. J. Kropf , F. Marmottini , M. Nocchetti , E. C. Wegener , F. Zaccaria , M. Delferro , R. Vivani , A. Macchioni , ACS Appl. Mater. Interfaces 2020, 12, 32736. 32583657 10.1021/acsami.0c07925 PMC8008397

[78] J. Park , Y. J. Sa , H. Baik , T. Kwon , S. H. Joo , K. Lee , ACS Nano 2017, 11, 5500. 28599106 10.1021/acsnano.7b00233

[79] S. M. Alia , S. Shulda , C. Ngo , S. Pylypenko , B. S. Pivovar , ACS Catal. 2018, 8, 2111.

[80] Y. Pi , Q. Shao , P. Wang , J. Guo , X. Huang , Adv. Funct. Mater. 2017, 27, 1700886.

[81] F. Lv , W. Zhang , W. Yang , J. Feng , K. Wang , J. Zhou , P. Zhou , S. Guo , Small Methods 2020, 4, 1900129.

[82] J. Zhu , Z. Chen , M. Xie , Z. Lyu , M. Chi , M. Mavrikakis , W. Jin , Y. Xia , Angew. Chem., Int. Ed. 2019, 58, 7244. 10.1002/anie.201901732 30848853

[83] Z. Fan , Z. Luo , Y. Chen , J. Wang , B. Li , Y. Zong , H. Zhang , Small 2016, 12, 3908. 27345872 10.1002/smll.201601787

[84] J. Feng , F. Lv , W. Zhang , P. Li , K. Wang , C. Yang , B. Wang , Y. Yang , J. Zhou , F. Lin , G.-C. Wang , S. Guo , Adv. Mater. 2017, 29, 1703798. 10.1002/adma.201703798 29083497

[85] F. Lv , J. Feng , K. Wang , Z. Dou , W. Zhang , J. Zhou , C. Yang , M. Luo , Y. Yang , Y. Li , P. Gao , S. Guo , ACS Cent. Sci. 2018, 4, 1244. 30276259 10.1021/acscentsci.8b00426 PMC6161040

[86] T. Kwon , M. Jun , G. J. Bang , H. Yang , J. Joo , T. Kim , J. Kim , J. M. Kim , H. Baik , Y. Jung , J. Y. Kim , K. Lee , Cell Rep. Phys. Sci. 2020, 1, 100260.

[87] J. Yin , J. Jin , M. Lu , B. Huang , H. Zhang , Y. Peng , P. Xi , C.-H. Yan , J. Am. Chem. Soc. 2020, 142, 18378. 32955265 10.1021/jacs.0c05050

[88] K.-R. Yeo , K.-S. Lee , H. Kim , J. Lee , S.-K. Kim , Energy Environ. Sci. 2022, 15, 3449.

[89] R. D. L. Smith , B. Sporinova , R. D. Fagan , S. Trudel , C. P. Berlinguette , Chem. Mater. 2014, 26, 1654.

[90] G. Li , S. Li , M. Xiao , J. Ge , C. Liu , W. Xing , Nanoscale 2017, 9, 9291. 28661529 10.1039/c7nr02899g

[91] J. Lim , D. Park , S. S. Jeon , C.-W. Roh , J. Choi , D. Yoon , M. Park , H. Jung , H. Lee , Adv. Funct. Mater. 2018, 28, 1704796.

[92] C. Zhao , H. Yu , Y. Li , X. Li , L. Ding , L. Fan , J. Electroanal. Chem. 2013, 688, 269.

[93] M. Faustini , M. Giraud , D. Jones , J. Rozière , M. Dupont , T. R. Porter , S. Nowak , M. Bahri , O. Ersen , C. Sanchez , C. Boissière , C. Tard , J. Peron , Adv. Mater. 2019, 9, 1802136.

[94] B. Jiang , J. Kim , Y. Guo , K. C. W. Wu , S. M. Alshehri , T. Ahamad , N. Alhokbany , J. Henzie , Y. Yamachi , Catal. Sci. Technol. 2019, 9, 3697.

[95] L. Zu , X. Qian , S. Zhao , Q. Liang , Y. E. Chen , M. Liu , B.-J. Su , K.-H. Wu , L. Qu , L. Duan , H. Zhan , J.-Y. Zhang , C. Li , W. Li , J. Y. Juang , J. Zhu , D. Li , A. Yu , D. Zhao , J. Am. Chem. Soc. 2022, 144, 2208. 35099956 10.1021/jacs.1c11241

[96] H. Su , X. Zhao , W. Cheng , H. Zhang , Y. Li , W. Zhou , M. Liu , Q. Liu , ACS Energy Lett. 2019, 4, 1816.

[97] T. Weber , J. Pfrommer , M. J. S. Abb , B. Herd , O. Khalid , M. Rohnke , P. H. Lakner , J. Evertsson , S. Volkov , F. Bertram , R. Znaiguia , F. Carla , V. Vonk , E. Lundgren , A. Stierle , H. Over , ACS Catal. 2019, 9, 6530.

[98] T. Weber , T. Ortmann , D. Escalera-López , M. J. S. Abb , B. Mogwitz , S. Cherevko , M. Rohnke , H. Over , ChemCatChem 2020, 12, 855.

[99] W. Xu , G. M. Haarberg , S. Sunde , F. Seland , A. P. Ratvik , S. Holmin , J. Gustavsson , Å. Afvander , E. Zimmerman , T. Åkre , Electrochim. Acta 2019, 295, 204.

[100] J. Shan , C. Guo , Y. Zhu , S. Chen , L. Song , M. Jaroniec , Y. Zheng , S.-Z. Qiao , Chem 2019, 5, 445.

[101] R. Huang , Y. Wen , P. Miao , W. Shi , W. Niu , K. Sun , Y. Li , Y. Ji , B. Zhang , Chem Catal. 2023, 3, 100667.

[102] Z. Shi , Y. Wang , J. Li , X. Wang , Y. Wang , Y. Li , W. Xu , Z. Jiang , C. Liu , W. Xing , J. Ge , Joule 2021, 5, 2164.

[103] C. T. K. Nguyen , N. Q. Tran , T. A. Le , H. Lee , Catalysts 2021, 11, 1176.

[104] H. N. Nong , H.-S. Oh , T. Reier , E. Willinger , M.-G. Willinger , V. Petkov , D. Teschner , P. Strasser , Angew. Chem., Int. Ed. 2015, 54, 2975. 10.1002/anie.201411072 25611732

[105] H.-S. Oh , H. N. Nong , T. Reier , A. Bergmann , M. Gliech , J. Ferreira de Araújo , E. Willinger , R. Schlögl , D. Teschner , P. Strasser , J. Am. Chem. Soc. 2016, 138, 12552. 27549910 10.1021/jacs.6b07199

[106] J. Gao , C.-Q. Xu , S.-F. Hung , W. Liu , W. Cai , Z. Zeng , C. Jia , H. M. Chen , H. Xiao , J. Li , Y. Huang , B. Liu , J. Am. Chem. Soc. 2019, 141, 3014. 30673269 10.1021/jacs.8b11456

[107] W. Q. Zaman , W. Sun , Z. Zhou , Y. Wu , L. Cao , J. Yang , ACS Appl. Energy Mater. 2018, 1, 6374.

[108] J. Cheng , J. Yang , S. Kitano , G. Juhasz , M. Higashi , M. Sadakiyo , K. Kato , S. Yoshioka , T. Sugiyama , M. Yamauchi , N. Nakashima , ACS Catal. 2019, 9, 6974.

[109] D. Lebedev , C. Copéret , ACS Appl. Energy Mater. 2019, 2, 196.

[110] X. Wen , L. Bai , M. Li , J. Guan , Mater. Today Energy 2018, 10, 153.

[111] Z. Wang , Z. Zheng , Y. Xue , F. He , Y. Li , Adv. Mater. 2021, 11, 2101138.

[112] P. Joshi , R. Yadav , M. Hara , T. Inoue , Y. Motoyama , M. Yoshimura , J. Mater. Chem. A 2021, 9, 9066.

[113] S. Choi , J. Park , M. K. Kabiraz , Y. Hong , T. Kwon , T. Kim , A. Oh , H. Baik , M. Lee , S.-M. Paek , S.-I. Choi , K. Lee , Adv. Funct. Mater. 2020, 30, 2003935.

[114] K. Sardar , S. C. Ball , J. D. B. Sharman , D. Thompsett , J. M. Fisher , R. A. P. Smith , P. K. Biswas , M. R. Lees , R. J. Kashtiban , J. Sloan , R. I. Walton , Chem. Mater. 2012, 24, 4192.

[115] K. Sardar , E. Petrucco , C. I. Hiley , J. D. B. Sharman , P. P. Wells , A. E. Russell , R. J. Kashtiban , J. Sloan , R. I. Walton , Angew. Chem., Int. Ed. 2014, 53, 10960. 10.1002/anie.201406668 PMC4497602 25196322

[116] J. Park , M. Risch , G. Nam , M. Park , T. J. Shin , S. Park , M. G. Kim , Y. Shao-Horn , J. Cho , Energy Environ. Sci. 2017, 10, 129.

[117] J. Parrondo , M. George , C. Capuano , K. E. Ayers , V. Ramani , J. Mater. Chem. A 2015, 3, 10819.

[118] M. A. Subramanian , G. Aravamudan , G. V. Subba Rao , Prog. Solid State Chem. 1983, 15, 55.

[119] D. Lebedev , M. Povia , K. Waltar , P. M. Abdala , I. E. Castelli , E. Fabbri , M. V. Blanco , A. Fedorov , C. Copéret , N. Marzari , T. J. Schmidt , Chem. Mater. 2017, 29, 5182.

[120] K. Ueda , T. Oh , B.-J. Yang , R. Kaneko , J. Fujioka , N. Nagaosa , Y. Tokura , Nat. Commun. 2017, 8, 15515. 28537276 10.1038/ncomms15515 PMC5458080

[121] Y. Tokiwa , J. J. Ishikawa , S. Nakatsuji , P. Gegenwart , Nat. Mater. 2014, 13, 356. 24651428 10.1038/nmat3900

[122] P.-C. Shih , J. Kim , C.-J. Sun , H. Yang , ACS Appl. Energy Mater. 2018, 1, 3992.

[123] C. Shang , C. Cao , D. Yu , Y. Yan , Y. Lin , H. Li , T. Zheng , X. Yan , W. Yu , S. Zhou , J. Zeng , Adv. Mater. 2019, 31, 1805104. 10.1002/adma.201805104 30549113

[124] W. Sun , J.-Y. Liu , X.-Q. Gong , W.-Q. Zaman , L.-M. Cao , J. Yang , Sci. Rep. 2016, 6, 38429. 27910932 10.1038/srep38429 PMC5133550

[125] B.-J. Kim , D. F. Abbott , X. Cheng , E. Fabbri , M. Nachtegaal , F. Bozza , I. E. Castelli , D. Lebedev , R. Schäublin , C. Copéret , T. Graule , N. Marzari , T. J. Schmidt , ACS Catal. 2017, 7, 3245.

[126] N.-I. Kim , Y. J. Sa , T. S. Yoo , S. R. Choi , R. A. Afzal , T. Choi , Y.-S. Seo , K.-S. Lee , J. Y. Hwang , W. S. Choi , S. H. Joo , J.-Y. Park , Sci. Adv. 2018, 4, eaap9360. 29951583 10.1126/sciadv.aap9360 PMC6018999

[127] J. Zhang , X. Zhao , L. Du , Y. Li , L. Zhang , S. Liao , J. B. Goodenough , Z. Cui , Nano Lett. 2019, 19, 7457. 31532687 10.1021/acs.nanolett.9b03168

[128] L. C. Seitz , C. F. Dickens , K. Nishio , Y. Hikita , J. Montoya , A. Doyle , C. Kirk , A. Vojvodic , H. Y. Hwang , J. K. Norskov , T. F. Jaramillo , Science 2016, 353, 1011. 27701108 10.1126/science.aaf5050

[129] L. Yang , L. Shi , H. Chen , X. Liang , B. Tian , K. Zhang , Y. Zou , X. Zou , Adv. Mater. 2023, 35, 2208539. 10.1002/adma.202208539 36586400

[130] C. W. Song , H. Suh , J. Bak , H. B. Bae , S.-Y. Chung , Chem 2019, 5, 3243.

[131] N. T. T. Thao , K. Kim , J. H. Ryu , B.-S. An , A. K. Nayak , J. U. Jang , K.-H. Na , W.-Y. Choi , G. Ali , K. H. Chae , M. Akbar , K. Y. Chung , H.-S. Cho , J. H. Park , B.-H. Kim , H. Han , Adv. Sci. 2023, 10, 2207695. 10.1002/advs.202207695 PMC10238205 36991522

[132] J. O. Bockris , J. Chem. Phys. 1956, 24, 817.

[133] J.-Y. Zhu , Q. Xue , Y.-Y. Xue , Y. Ding , F.-M. Li , P. Jin , P. Chen , Y. Chen , ACS Appl. Mater. Interfaces 2020, 12, 14064. 32125818 10.1021/acsami.0c01937

[134] L. Fu , F. Yang , G. Cheng , W. Luo , Nanoscale 2018, 10, 1892. 29313049 10.1039/c7nr09377b

[135] B. Jiang , Y. Guo , J. Kim , A. E. Whitten , K. Wood , K. Kani , A. E. Rowan , J. Henzie , Y. Yamauchi , J. Am. Chem. Soc. 2018, 140, 12434. 30129750 10.1021/jacs.8b05206

[136] G. Wu , X. Zheng , P. Cui , H. Jiang , X. Wang , Y. Qu , W. Chen , Y. Lin , H. Li , X. Han , Y. Hu , P. Liu , Q. Zhang , J. Ge , Y. Yao , R. Sun , Y. Wu , L. Gu , X. Hong , Y. Li , Nat. Commun. 2019, 10, 4855. 31649272 10.1038/s41467-019-12859-2 PMC6813339

[137] F. Luo , L. Guo , Y. Xie , J. Xu , K. Qu , Z. Yang , Appl. Catal. B 2020, 279, 119394.

[138] J. Shan , T. Ling , K. Davey , Y. Zheng , S.-Z. Qiao , Adv. Mater. 2019, 31, 1900510. 10.1002/adma.201900510 30811671

[139] H. Guo , Z. Fang , H. Li , D. Fernandez , G. Henkelman , S. M. Humphrey , G. Yu , ACS Nano 2019, 13, 13225. 31668069 10.1021/acsnano.9b06244

[140] T. Kwon , H. Hwang , Y. J. Sa , J. Park , H. Baik , S. H. Joo , K. Lee , Adv. Funct. Mater. 2017, 27, 1604688.

[141] Z. Jin , J. Lv , H. Jia , W. Liu , H. Li , Z. Chen , X. Lin , G. Xie , X. Liu , S. Sun , H.-J. Qiu , Small 2019, 15, 1904180. 10.1002/smll.201904180 31596058

[142] Y. Pi , J. Guo , Q. Shao , X. Huang , Chem. Mater. 2018, 30, 8571.

[143] Z. Zhuang , Y. Wang , C.-Q. Xu , S. Liu , C. Chen , Q. Peng , Z. Zhuang , H. Xiao , Y. Pan , S. Lu , R. Yu , W.-C. Cheong , X. Cao , K. Wu , K. Sun , Y. Wang , D. Wang , J. Li , Y. Li , Nat. Commun. 2019, 10, 4875. 31653856 10.1038/s41467-019-12885-0 PMC6814841

[144] W. Gou , M. Zhang , Y. Zou , X. Zhou , Y. Qu , ChemCatChem 2019, 11, 6008.

[145] D. Weber , L. M. Schoop , D. Wurmbrand , S. Laha , F. Podjaski , V. Duppel , K. Müller , U. Starke , B. V. Lotsch , J. Mater. Chem. A 2018, 6, 21558.

[146] G. Meng , W. Sun , A. A. Mon , X. Wu , L. Xia , A. Han , Y. Wang , Z. Zhuang , J. Liu , D. Wang , Y. Li , Adv. Mater. 2019, 31, 1903616. 10.1002/adma.201903616 31373731

[147] J. Zhu , Z. Lyu , Z. Chen , M. Xie , M. Chi , W. Jin , Y. Xia , Chem. Mater. 2019, 31, 5867.

[148] W. Q. Zaman , W. Sun , M. Tariq , Z. Zhou , U. Farooq , Z. Abbas , L. Cao , J. Yang , Appl. Catal., B 2019, 244, 295.

[149] L. Yang , G. Yu , X. Ai , W. Yan , H. Duan , W. Chen , X. Li , T. Wang , C. Zhang , X. Huang , J.-S. Chen , X. Zou , Nat. Commun. 2018, 9, 5236. 30531797 10.1038/s41467-018-07678-w PMC6286314

[150] L. Yang , H. Chen , L. Shi , X. Li , X. Chu , W. Chen , N. Li , X. Zou , ACS Appl. Mater. Interfaces 2019, 11, 42006. 31633901 10.1021/acsami.9b11287

[151] X. Liang , L. Shi , Y. Liu , H. Chen , R. Si , W. Yan , Q. Zhang , G.-D. Li , L. Yang , X. Zou , Angew. Chem. 2019, 131, 7713. 10.1002/anie.201900796 30775830

[152] S. Y. Jung , K. M. Kim , J. H. Ryu , S. Yeo , H. Jeon , A. K. Nayak , N. Thi Thu Thao , E. Enkhtuvshin , S. J. Kim , J. U. Jang , M. G. Kim , K.-H. Na , W.-Y. Choi , J. Bang , S. Choi , T. Song , S. Mhin , H. Han , J. Alloys Compd. 2023, 946, 169466.

